# Prediction models for incident stroke in the community: a systematic review and meta-analysis of predictive performance

**DOI:** 10.1093/ehjdh/ztaf147

**Published:** 2026-02-05

**Authors:** Mohammad Haris, Elizabeth Romer, Tanina Younsi, Jianhua Wu, Harriet Larvin, Chris Wilkinson, Alan Cameron, Giulio F Romiti, Gregory Y H Lip, Ramesh Nadarajah, Chris P Gale

**Affiliations:** Leeds Institute for Cardiovascular and Metabolic Medicine, University of Leeds, 6 Clarendon Way, Leeds, LS2 9DA, UK; Leeds Institute of Data Analytics, University of Leeds, Clarendon Way, Leeds, LS2 9NL, UK; Department of Cardiology, Leeds Teaching Hospitals NHS Trust, Great George Street, Leeds, LS1 3EX, UK; Department of Cardiology, Airedale NHS Foundation Trust, Keighley, UK; Department of Cardiology, Leeds Teaching Hospitals NHS Trust, Great George Street, Leeds, LS1 3EX, UK; Wolfson Institute of Population Health, Queen Mary University of London, London, UK; Wolfson Institute of Population Health, Queen Mary University of London, London, UK; Hull York Medical School, University of York, York, UK; Academic Cardiovascular Unit, South Tees NHS Foundation Trust, James Cook University Hospital, Middlesbrough, UK; School of Cardiovascular and Metabolic Health, University of Glasgow, Glasgow, UK; Department of Translational and Precision Medicine, Sapienza - University of Rome, Rome, Italy; Liverpool Centre for Cardiovascular Science, at University of Liverpool, Liverpool John Moores University and Liverpool Heart & Chest Hospital, Liverpool, United Kingdom; Liverpool Centre for Cardiovascular Science, at University of Liverpool, Liverpool John Moores University and Liverpool Heart & Chest Hospital, Liverpool, United Kingdom; Danish Center for Health Services Research, Department of Clinical Medicine, Aalborg University, Aalborg, Denmark; Department of Cardiology, Lipidology and Internal Medicine with Intensive Coronary Care Unit, Medical University of Bialystok, Bialystok, Poland; Leeds Institute for Cardiovascular and Metabolic Medicine, University of Leeds, 6 Clarendon Way, Leeds, LS2 9DA, UK; Leeds Institute of Data Analytics, University of Leeds, Clarendon Way, Leeds, LS2 9NL, UK; Department of Cardiology, Leeds Teaching Hospitals NHS Trust, Great George Street, Leeds, LS1 3EX, UK; Leeds Institute for Cardiovascular and Metabolic Medicine, University of Leeds, 6 Clarendon Way, Leeds, LS2 9DA, UK; Leeds Institute of Data Analytics, University of Leeds, Clarendon Way, Leeds, LS2 9NL, UK; Department of Cardiology, Leeds Teaching Hospitals NHS Trust, Great George Street, Leeds, LS1 3EX, UK

**Keywords:** stroke, Cerebrovascular disease, Prediction, Prevention, Community

## Abstract

**Aims:**

Stroke is the second leading cause of death and the third leading cause of disability worldwide. We performed a systematic review and meta-analysis of multivariable models applicable to the prediction of incident stroke in community cohorts.

**Methods and results:**

Ovid Medline and Embase were searched for studies related to stroke and prediction models from inception to 3 November 2025. Measures of discrimination were extracted and pooled by Bayesian meta-analysis, with heterogeneity assessed through a 95% prediction interval (PI). Risk of bias was assessed using the Prediction model Risk Of Bias Assessment Tool and certainty in effect estimates by Grading of Recommendations, Assessment, Development and Evaluation. Forty-one studies met the inclusion criteria, describing 80 prediction models, with two (R-FSRS and Basic IS) eligible for meta-analysis, including 969 514 participants. Both R-FSRS (summary c-statistic 0.714, 95% CI 0.681–0.747) and Basic IS (0.709, 95% CI 0.647–0.769) showed acceptable discrimination performance. Risk of bias was high in 66% of models, and both models showed reduced performance when excluding development cohorts and studies at high risk of bias (R-FSRS, 0.667, 95% CI 0.604–0.727; Basic IS 0.701; 95% CI 0.583–0.807). Only 43% of studies reported calibration, and no model underwent clinical utility analysis or a clinical impact study.

**Conclusion:**

Many models have been derived for stroke prediction, however, they are rarely externally validated, and studies are limited by a high risk of bias, poor reporting of calibration and a lack of clinical utility analysis or prospective validation. Thus, the evidence base is insufficient to translate these models to clinical practice.

## Graphical Abstract

**Graphical Abstract:**
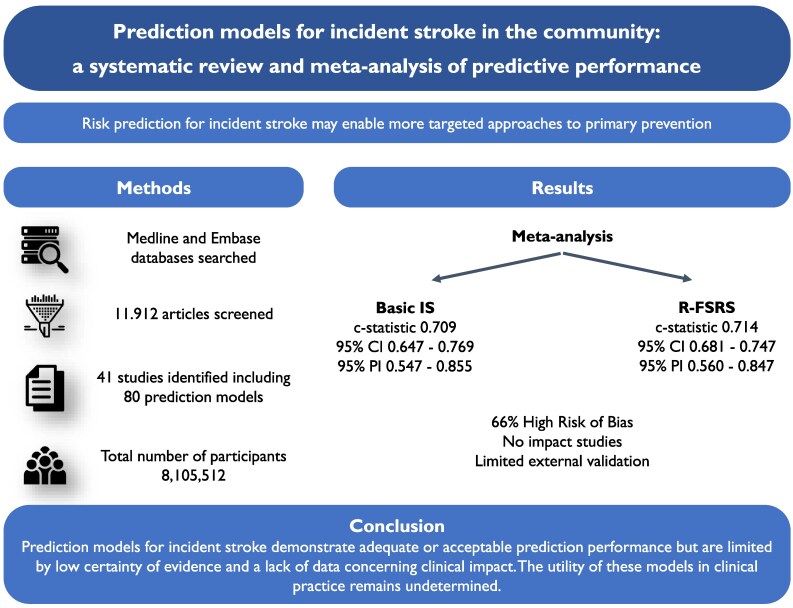

Key Learning Points **What is already known:**

- Stroke is a leading global cause of death and disability, with prevention strategies emphasising risk prediction tools.
- Several multivariable models for stroke prediction exist, but many include variables not routinely collected or are developed in specific subpopulations.
- Previous systematic reviews have not clearly evaluated which models perform best in the general population.

**What this study adds:**

- This is a comprehensive review of stroke prediction models that use routinely available variables.
- Only two models (Revised Framingham Stroke Risk Score and Basic Ischaemic Stroke) were suitable for meta-analysis, showing acceptable performance, which reduced when studies at high risk of bias and derivation cohorts were excluded.
- No studies reported clinical utility or real-world impact, highlighting an urgent need for external validation and impact studies before clinical implementation.

## Introduction

The absolute number of cases of stroke continues to rise due to an ageing population.^1^ Stroke is already the second leading cause of death worldwide, responsible for almost seven million deaths annually,^2,3^ and the burden of stroke-related death is expected to increase by 50% in the coming decades.^4,5^ Moreover, stroke is the third leading cause of adult disability worldwide.^2,3^ Accordingly, it carries a significant healthcare and societal cost burden, accounting for approximately 34% of total global healthcare expenditure^6,7^ and costing in excess of US $891 billion annually.^5^

While advancements in reperfusion therapies, dedicated stroke unit care, and early rehabilitation have conferred significant improvements in outcomes, prevention remains the optimal approach to reduce the burden of stroke.^8,9^ Risk prediction for incident stroke may enable more targeted approaches to primary prevention. Many risk prediction tools have been derived to predict the composite risk of cardiovascular disease overall,^10,11^ and stroke specifically.^12–14^ Guidelines for the primary prevention of stroke emphasize the use of these risk prediction tools to aid in employing effective strategies to prevent incident stroke.^8,15–17^

However, the clinical utility of existing stroke prediction tools in the general population remains uncertain, as no prior evidence specifically addresses models applicable to this setting. Previous systematic reviews have included specific subpopulations not reflective of the general population, included composite scores which often do not give a specific risk of developing stroke, or included scores with variables not readily available in the community.^18–24^ To address this knowledge gap, we performed a systematic review of prediction models for incident stroke in the general population and conducted a quantitative synthesis of predictive performance.

## Methods

This systematic review is registered on PROSPERO (CRD42024511343) and has been reported in accordance with the Preferred Reporting Items for Systematic Reviews and Meta-Analyses (PRISMA) guidelines (see Supplementary material).

### Search strategy and inclusion criteria

The CHecklist for critical Appraisal and data extraction for systematic Reviews of prediction Modelling Studies (CHARMS) was used to frame the research question (see Supplementary material). The Ovid Medline and Ovid Embase databases were searched from inception to 3 November 2025 using a combination of keywords and subject headings related to stroke and prediction models (see Supplementary material), restricted to human studies. The full search strategy can be found in the Supplementary material. We performed forward and backward citation searches and reviewed previous systematic reviews. We used Endnote’s duplicate identification tool and then manually removed all remaining duplicates.

Articles were included if they were an original study in adults (≥18 years of age), developed and/or validated a prediction model for incident stroke based on multivariable analysis, provided a prediction performance metric for discrimination performance for a stroke outcome and were written in English. Articles were excluded if they included patients with stroke at baseline, only reported measures of association between risk factors and incident stroke rather than a full prediction model, studied only a subset of the general population (for example, individuals with a particular morbidity), or incorporated variables that would not be routinely available with high completeness in community health records (e.g. Townsend score, waist circumference, exercise, diet, uric acid, left ventricular hypertrophy; Supplementary material). Where studies reported multiple models, only those utilising variables readily obtainable in community settings were included.

We uploaded records to a systematic review web application (Rayyan, Qatar Computing Research Institute).^25^ Three investigators (M.H., E.R. and T.Y.) independently screened them for inclusion by title, abstract, full text and Supplementary materials. Disagreements were resolved by consultation with a fourth investigator (R.N.).

### Data extraction and quality assessment

Two investigators (M.H. and E.R.) independently extracted the data from the included studies based on the CHecklist for critical Appraisal and data extraction for systematic Reviews of prediction Modelling Studies (CHARMS). All data came from the primary reference, unless otherwise stated.

To allow quantitative synthesis and assessment of the predictive performance of the models, we extracted measures of discrimination and calibration.^26^ Discrimination assesses the model's ability to differentiate between individuals who will experience the outcome and those who will not. To assess discrimination, we extracted data on the c-statistic or the area under the receiver operating characteristic (AUROC), along with their corresponding 95% confidence intervals (CI). If the reported CI was missing, we computed it using the methods outlined by Debray *et al*.^26^ Calibration evaluates the accuracy of the model's predicted probabilities, and we extracted all performance measures reported. Three investigators (M.H., E.R. and T.Y.) assessed the models for risk of bias and applicability to our review question using the Prediction model Risk Of Bias ASsessment Tool (PROBAST).^27^ We also checked for reporting of the clinical utility of a model (net benefit in the form of decision curve analysis or decision analytical modelling, which can be used to integrate the benefits and harms of using a model for clinical decision support) and conducted forward citation searching for studies determining the impact (clinical and cost-effectiveness) of using models in real world clinical practice.

### Data synthesis and statistical analysis

We reported continuous variables as means ± standard deviation (SD) and categorical variables as percentages. We evaluated statistical significance in all analyses at the 0.05 level. When a study reported on multiple cohorts and presented separate data for each cohort, we assessed model performance separately for each cohort within that study. A funnel plot was created to check for publication bias.^28^

We conducted a Bayesian meta-analysis of discrimination through a summary measure of c-statistic and corresponding 95% CI. We calculated the 95% prediction interval (PI) to depict the extent of between-study heterogeneity and to indicate a possible range for prediction model performance in a new validation.^29^ A PI is a statistical measure to estimate a range for the predicted model performance in a new validation of the model with a certain level of confidence. Summary c-statistics of <0.60, 0.60–0.69, 0.70–0.80, and >0.80 were defined *a priori* as inadequate, adequate, acceptable and good based on prior publications.^30,31^

All Bayesian meta-analysis models assume random effects by default. Results are based on the posterior median. PIs are directly obtained from the corresponding posterior quartiles. The standard model for random effects meta-analysis assumes that the ‘true’ performance is normally distributed within and across studies.^32^ Within-study normality of performance estimates can be justified with this selection of included studies because they are all large. Snell *et al*. showed that the between-study distribution of the c-statistic on the original scale is not normally distributed when there is variability in the predictor effect across studies (which is likely in this selection of studies, as they include different populations and adopt slightly different definitions for predictors).^32^ They found that the logit scale is more appropriate for the estimation of the PI. Consequently, we used the ‘valmeta’ function of the ‘metamisc’ package in R software, which applies a logit transformation to the c-statistic prior to calculation of the summary c-statistic and PI.^33–35^

For appropriate prior distributions, we borrowed from earlier work by Debray *et al.,* which recommended a half Student-*t* distribution with location m, scale σ, and v degrees of freedom, where we set m = 0 and define σ equal to the largest empirical value of 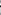 (to allow for more extreme values of heterogeneity).^29^ These hyperparameter values allow to penalize the extent of between-study heterogeneity when the number of included validation studies is low.^29^ Further, we also used v = 3 to ensure that the variance σ^2^ − v/(v − 2) exists and samples of τ were truncated above 10 to rule out unreasonable values. Thus, the resulting priors are given as τdiscr ∼ Student−*t*(0, 0.5^2^, 3)T[0.10], which has been shown to allow for large but realistic values for between-study heterogeneity.^29^

Our primary analysis assessed overall discrimination for models that had three or more cohorts with c-statistic data, excluding studies with a high risk of bias. We performed sensitivity analysis in which we assessed discrimination for each model, excluding results from derivation cohorts. We performed a further analysis comparing models employing regression techniques vs. machine learning techniques.

## Results

### Study selection

**Figure 1:**
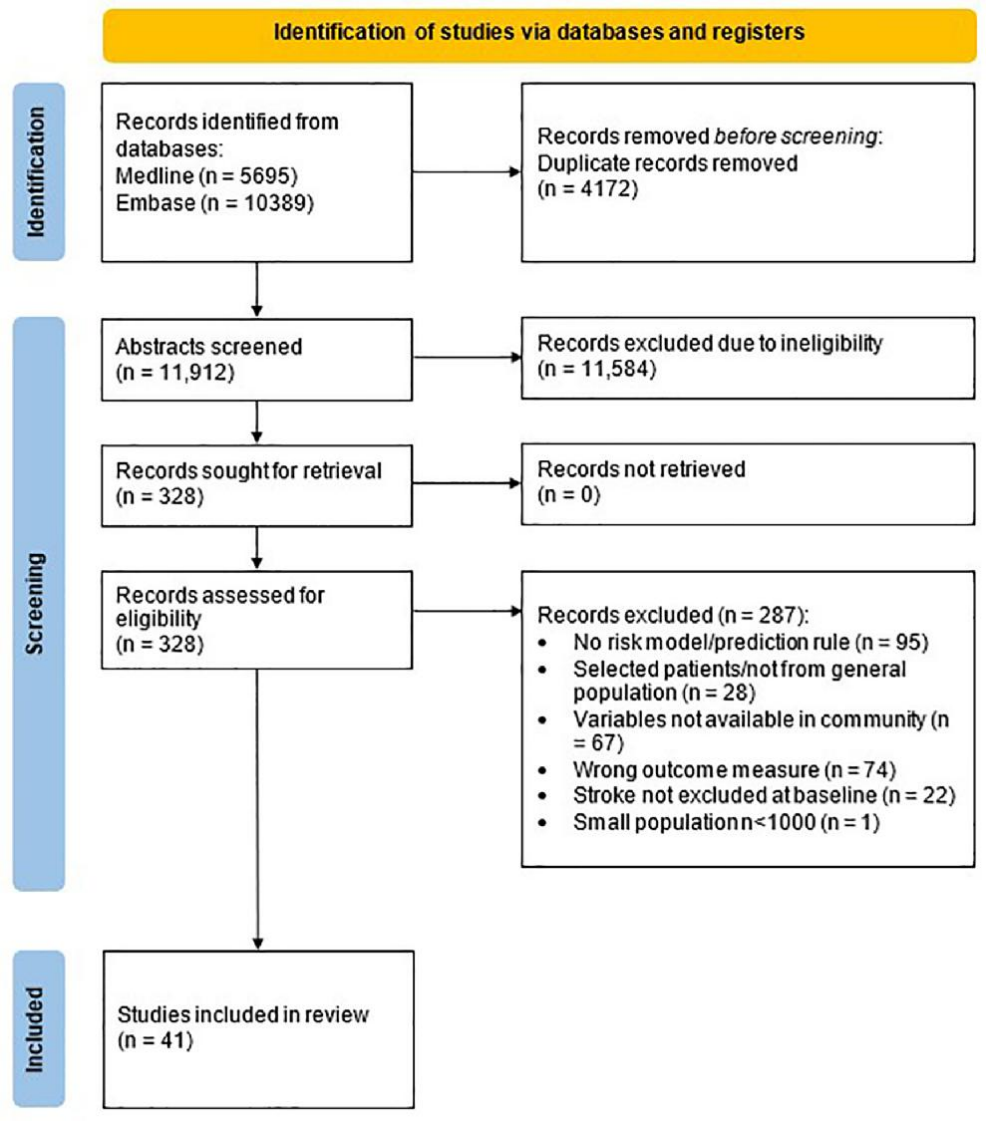
Flow diagram of literature search.

We identified 11 912 unique records, reviewed 328 full-text reports and included 41 studies (*Figure 1*). A list of excluded studies that met a number of the inclusion criteria is available in the Supplementary material.

### Characteristics of included studies

In the 41 studies, 80 models were derived and/or validated across 48 cohorts from a range of countries located in Asia Pacific (*n* = 21), the United States (*n* = 14) and Europe (*n* = 13) (*Table 1*). The total number of participants included in the studies was 8 105 512, with cohort sizes ranging from 1131 to 1 730 828. The mean age ranged from 35.5 to 68.6 years, and the proportion of women ranged from 0.0% to 66.1% (see Supplementary material online, *Table S1*).

**Table 1:** Overview of included studies

Study	Cohort (country)	Study aim	Stroke cases (*n*)/total patients (*n*) (%)	Outcome definition	Enrolment period (mean F/U in years)	Exclusion criteria	Overall ROB
Arafa *et al.* 2021^36^	Suita (Japan)	D, IV	372/6641 (5.6)	Cerebral infarction, ICH, SAH	1989–1999 (17.1)	History of cardiovascular diseases	High
						Age >79 or <30 y, Missing data	
Assman *et al.* 2007^37^	PROCAM (Germany)	D	85/8130 (1)	Focal neurological deficit >24 h due to vascular event	1978–1995 (12)	Haemorrhagic or undetermined stroke	High
Borglykke *et al.* 2010^38^	MORGAM (Denmark)	D, IV	2928/88290 (3.3)	Fatal and non-fatal stroke	1982–1997	History of MI/stroke	High
						Missing data	
Camen *et al.* 2020^39^	MORGAM (Denmark)	EV	3033/82881 (3.7)	Ischaemic/haemorrhagic/Indeterminate	1982–2010 (12.7)	History of MI/stroke/CAD	High
Chambless *et al.* 2004^40^	ARIC (US)	D	434/14685 (3)	Ischaemic	1987–1989 (12.3)	History of MI/stroke/CAD	High
						Race other than black or white	
						Missing data	
Chien *et al.* 2010^41^	Chin-Shan (Taiwan)	D, IV, EV	240/3512 (6.8)	Ischaemic/haemorrhagic	1990–2007 (15.9 median)	History of stroke/TIA	High
Chun *et al.* 2022^42^	CKB (China)	D, EV	43234/503842 (8.6)	Ischaemic/haemorrhagic	2004–2008 (9)	—	High
Di Castelnuovo *et al.* 2019^43^	BiomarCaRE (Finland, Sweden, Ireland, France, Italy)	D	1550/58173 (2.7)	Ischaemic/haemorrhagic	—	History of stroke	High
Ferket *et al.* 2014^44^	ARIC (US)	D	2884/27493 (10.5)	Ischaemic/haemorrhagic	—	History of stroke/AF	Low
	Rotterdam (Netherlands)					Current anticoagulation	
						Not Caucasian/African American	
	CHS (US)						
Flueckiger *et al.* 2018^45^	MESA (US)	EV	231/6712 (3.4)	Ischaemic or haemorrhagic	—	ABI >1.4	High
Foraker *et al.* 2016^46^	Jackson Heart Study (US)	EV	112/4140 (2.7)	Ischaemic or haemorrhagic	—	History of stroke	Low
						Missing data	
Harada *et al.* 2018^47^	JALS (Japan)	D	1351/67969 (2)	Ischaemic or haemorrhagic	2002–2004 (6.9)	History of stroke or heart disease	High
						Age >90 or <40 y, Missing data	
Hilvo *et al.* 2022^48^	FINRISK 2002 (Finland)	D	249/7810 (3.2)	Ischaemic or haemorrhagic	—	SAH	High
Hong *et al.* 2023^49^	FOS, ARIC, MESA, REGARDS (US)	EV	2199/62482 (3.5)	Ischaemic, haemorrhagic or other stroke	1971–2008 (10)	—	Low
Howard *et al.* 2017^50^	REGARDS (US)	D, EV	939/23983 (3.9)	Stroke	2003–2007 (8.2)	—	High
Hung *et al.* 2019^51^	NHIRD (Taiwan)	D, IV	2544/840487 (0.3)	Ischaemic stroke	2003–2003 (8)	—	Low
Hung *et al.* 2018^52^	NHIRD (Taiwan)	D, IV	4795/552898 (0.9)	Ischaemic stroke	2003–2003 (5)	—	High
Hunter *et al.* 2022^53^	FHS, FOS, FHS-3rd gen, FHS-OMNI2, FHS new offspring (US)	D, IV	14983/113714 (13.2)	Ischaemic stroke/TIA	1948–2018 (5)	Haemorrhagic stroke	Low
Jung *et al.* 2018^54^	KCPS-II (South Korea)	D, IV	823/144594 (5.7)	Ischaemic or haemorrhagic	2004–2005	—	High
Lee *et al.* 2020^55^	NHIS, NHIS-NSC (South Korea)	D, IV	3484/973055 (0.4)	Ischaemic, haemorrhagic or other stroke	2010–2011	—	High
Li X *et al.* 2022^56^	Jilin University Cohorts 1–3 (China)	D, IV	15833/32366 (49)	Ischaemic stroke	2018–2021 (5)	—	Low
Li Y *et al.* 2022 (1)^57^	CPRD (UK)	D, IV	224442/1730828 (12.9)	Ischaemic, haemorrhagic or any stroke	—	—	High
Lolak *et al.* 2023^58^	Ramathibodi Hospital (Thailand)	D, IV	9659/275247 (3.5)	Ischaemic or haemorrhagic	2010–2020	History of stroke	High
						<2 visits during period	
Majed *et al.* 2013^59^	PRIME (combined, France, Ireland)	D, IV	138/9638 (1.4)	First stroke event	1991–1993 (10)	History of stroke or CAD	Low
						Missing data	
	Framingham (US)						
Marrugat *et al.* 2014^60^	FRESCO Study (Spain)	D, EV	786/50408 (1.6)	Ischaemic or haemorrhagic	1992–2005 (9.3)	History of cardiovascular disease	Low
Teoh *et al.* 2018^61^	TJTC Hospital (Japan)	D, IV	2725/8175 (33.3)	First stroke	2001–2015	History of stroke/MI/HF	High
						Age <45 or >94 years	
Wannamethee *et al.* 2005^62^	BRHS (UK)	D, IV	291/5128 (5.7)	Stroke	1978–2000 (21.3)	History of stroke/coronary heart disease/diabetes	High
Wu *et al.* 2020^63^	CLHLS (China)	D, IV	56/1131 (5)	Stroke	2012–2012 (2)	History of stroke	Low
						Age <60 years	
						Missing data	
Yang *et al.* 2023^64^	CHARLS (China)	D, IV	312/5844 (5.3)	Stroke	2011–2011 (7)	Age <45 years	Low
Yatsuya *et al.* 2016^65^	JPHC I, JPHC II (Japan)	D, IV, EV	863/27270 (3.2)	Ischaemic or haemorrhagic	1990–1994 (16.4)	History of stroke/MI	Low
						Missing data	
Yatsuya *et al.* 2013^66^	JPHC I, JPHC II (Japan)	D, IV, EV	790/15672 (5)	Ischaemic or haemorrhagic	1990–1994 (14)	History of cardiovascular disease	Low
						Missing data	
Zhang X *et al.* 2005^67^	Beijing steelworker cohort (China)	D, IV	118/4400 (2.7)	Ischaemic or haemorrhagic	1974–1980 (13.5)	Age <35 years	High
Zhang Y *et al.* 2020^68^	BLSA (China)	D, EV	106/1203 (8.8)	Stroke	2009–2009 (4.8)	History of stroke	Low
						Age >84 years	
						Missing data	
Dufouil *et al.* 2017^69^	FHS, REGARDS, 3C (US)	D, EV	974/36364 (2.7)	Ischaemic or haemorrhagic	1948–2007	History of stroke	Low
						Age <55 or >84 years	
Hung *et al.* 2017^70^	NHIRD (Taiwan)	D, IV	4944/798611 (0.6)	Ischaemic stroke	2003–2003 (5)	History of stroke	High
						Missing data	
Sun *et al.* 2021^71^	UKB, CPRD (UK)	D, IV	2347/306654 (0.8)	Ischaemic or haemorrhagic	2006–2010 (8.1)	History of stroke/TIA/heart disease/peripheral vascular disease, taking lipid-lowering drugs, and Missing data	High
Xing *et al.* 2019^72^	China MUCA, CIMIC (China)	EV	2690/84961 (3.2)	Ischaemic or haemorrhagic	MUCA 1992–1994 (17.1)	History of cardiovascular disease Age <35 or >74 years	High
					CIMIC 2007–2008 (5.9)		
Bos *et al.* 2017^73^	Rotterdam (Netherlands)	EV	502/7966 (6.3)	Ischaemic or haemorrhagic	2000–2006 (7)	History of cardiovascular disease/AF	High
D’Agostino *et al.* 2008^74^	FHS, FOS (US)	D, IV, EV	177/8491 (2)	Ischaemic/haemorrhagic/TIA	1968–1987 (12)	History of cardiovascular disease	Low
						Age <30 or >74 y, Missing data	
Li Y *et al.* 2022 (2)^75^	CPRD (UK)	D, IV, EV	74547/1096275 (6.8)	Ischaemic/haemorrhagic/TIA	1985–2015 (5)	Missing data	Low
Vu *et al.* 2024^76^	Suita (Japan)	D	438/7389 (5.9)	Ischaemic or haemorrhagic	1989–1999 (15)	History of cardiovascular diseases	High
						Age >79 or <30 y, Missing data	

ACVD, atherosclerotic cardiovascular disease; ADA, Active Data Augmenter; AF, atrial fibrillation; ARIC, Atherosclerosis Risk In Communities; BLSA, Beijing Longitudinal Study of Aging; BN, Bayesian Network; BRHS, British Regional Heart Study; CAD, coronary artery disease; CHARLS, China Health and Retirement Longitudinal Study; CHS, Cardiovascular Health Study; CIMIC, Community Intervention of Metabolic Syndrome in China; CKB, China Kadoorie Biobank; CLHLS, Chinese Longitudinal Healthy Longevity Survey; CNN, Convolutional Neural Network; CORSAIB, COR Sà Illes Balears; CPRD, Clinical Practice Research Datalink; CVD, cardiovascular disease; D, derivation; DNN, Deep Neural Network; EV, external validation; FHS, Framingham Heart Study; FOS, Framingham Offspring Study; FRESCO, (Función de Riesgo ESpañola de acontecimientos Coronarios y Otros; FSRS, Framingham Stroke Risk Score; GBDT, Gradient-Boosted Decision Tree; GRU, Gated Recurrent Unit; HF, heart failure; ICH, intracerebral haemorrhage; IHD, ischaemic heart disease; IS, ischaemic stroke; IV, internal validation; JALS, Japan Arteriosclerosis Longitudinal Study; JPHC, Japan Public Health Center; LR, Logistic Regression; LVH, left ventricular hypertrophy; MESA, Multi-Ethnic Study of Atherosclerosis; MGL, Multiple Group Learning; MI, myocardial infarction; MORGAM, MOnica Risk, Genetics, Archiving and Monograph; NHIRD, National Health Insurance Research Database; NHIS, National Health Insurance Service; PCE, Pooled Cohort Equation; PRIME, Prospective Epidemiological Study of Myocardial Infarction; PROCAM, Prospective Cardiovascular Münster; R-FSRS, Revised Framingham Stroke Risk Score; REGARDS, Reasons for Geographic and Racial Differences in Stroke; RF, Random Forest; RLR, Relational Logistic Regression; RNN, Recurrent Neural Network; SAH, subarachnoid haemorrhage; SDA, Straightforward Data Augmentation; SRSRF, Self-Reported Stroke Risk Stratification; SVM, Support Vector Machine; TAN, Tree-Augmented Naïve Bayes; TIA, transient ischaemic attack; TJTC, Tsuyama Jifukai Tsuyama Chuo; TRS, Traditional Risk Score; XG Boost, extreme gradient boosting

### Characteristics of included prediction models

The 80 included models consisted of 51 multivariable regression models and 29 machine learning models. All studies reported the predictors in the models and prediction horizons ranged from one to 20 years. The range of reported AUROC was between 0.54 and 0.99, and 15 of the models were externally validated (see Supplementary material online, *Table S2*). Twenty-one models only predicted ischaemic strokes, and 59 models predicted both ischaemic and haemorrhagic strokes (see Supplementary material online, *Table S3*). The machine learning models were all supervised machine learning and included gradient boosting, logistic regression, random forest, support vector machine, neural networks and XGBoost (see Supplementary material online, *Table S4*).

Only 18 studies reported calibration outcomes. Of these, 10 reported Hosmer-Lemeshow statistic, 4 reported O:E ratio and 11 reported calibration plots.

**Figure 2:**
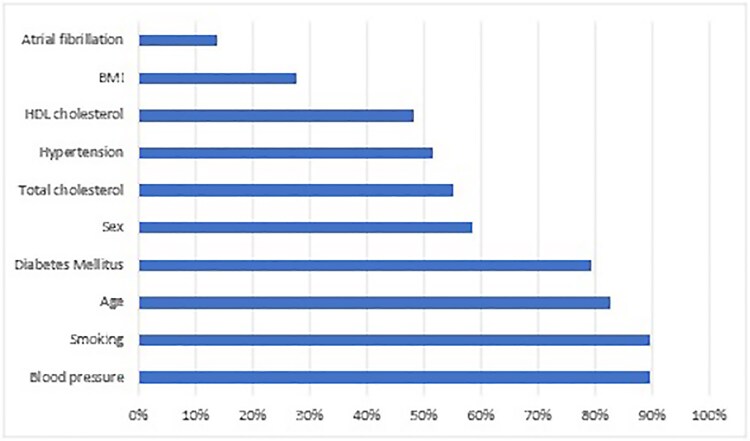
An overview of the 10 predictors most frequently incorporated in the prediction models in this study. HDL, high-density lipoprotein; BMI, body mass index.

The most common predictors in the regression models were blood pressure (BP) (89.7%), smoking (89.7%), age (82.8%) and diabetes mellitus (79.3%), as shown in *Figure 2*. Other variables included physical measurements such as BP and body mass index (BMI), medical conditions such as hypertension and atrial fibrillation, investigations such as blood glucose and cholesterol, and medications such as antihypertensives. Machine learning and regression-based models used broadly similar sets of predictors, and their discrimination performance was comparable (see Supplementary material online, *Tables S2*, *S5* and *S6*).

### Clinical utility and clinical impact of included models

No studies conducted a clinical utility analysis, and forward citation searching did not identify studies of clinical impact for the included risk prediction models.

### Risk of bias assessment

**Figure 3:**
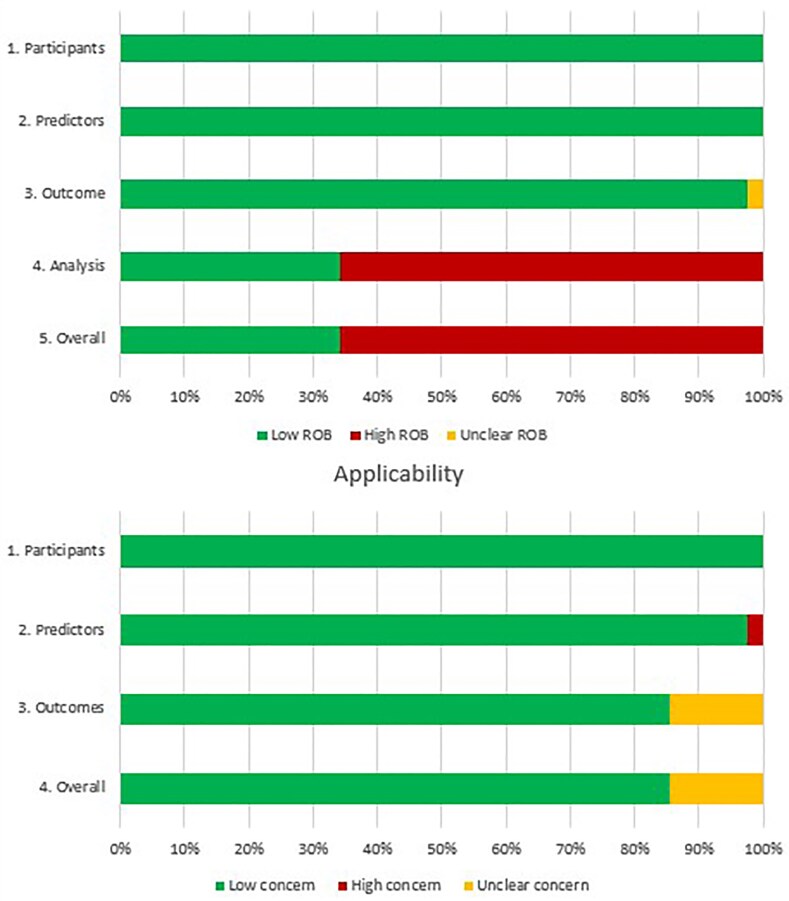
Judgements on the four prediction model risk of bias assessment tool (PROBAST) risk of bias domains and three PROBAST applicability domains presented as percentages across all included studies.

Overall, 66% of model results were at high risk of bias solely driven by high risk of bias in the analysis domain, mainly due to the handling of missing data (*Figure 3*, Supplementary material online, *Table S7*).

### Meta-analysis

**Figure 4:**
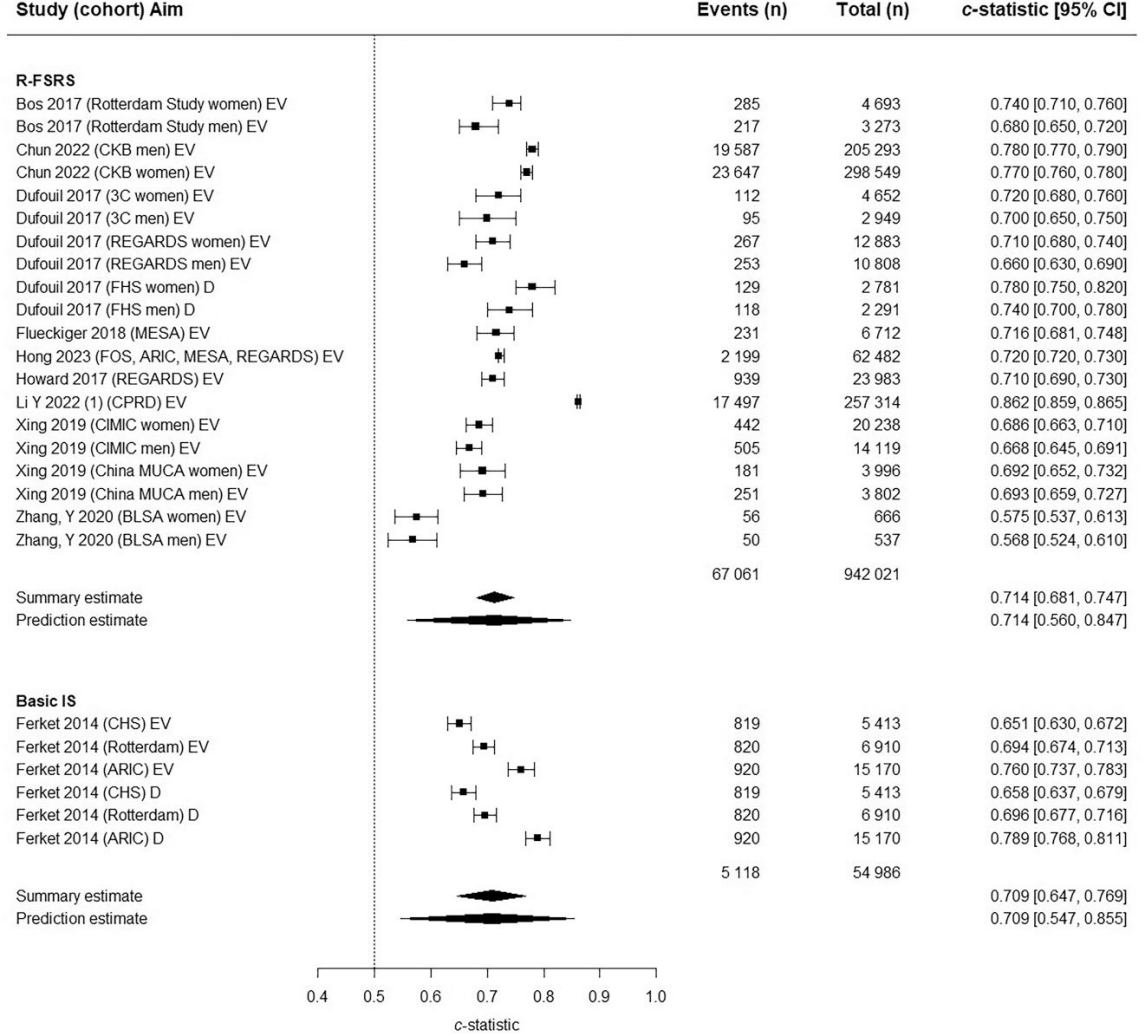
Forest plot of primary analysis of *c-*statistics. ARIC, Atherosclerosis Risk in Communities; BLSA, Beijing Longitudinal Study of Ageing; CHS, Cardiovascular Health Study; CIMIC, Community Intervention of Metabolic Syndrome in China; CKB, China Kadoorie Biobank; CPRD, Clinical Practice Research Datalink; D, Derivation; EV, External Validation; FHS, Framingham Heart Study; ICH, intracerebral haemorrhage; IS, ischaemic stroke; MESA, Multi-Ethnic Study of Atherosclerosis; REGARDS, Reasons for Geographic and Racial Differences in Stroke; R-FSRS, Revised Framingham Stroke Risk Score.

The Revised Framingham Stroke Risk Score (R-FSRS) and Basic Ischaemic Stroke (Basic IS) models were eligible for the primary meta-analysis, which included the derivation and validation cohorts and incorporated a total of 969 514 participants. Both models had acceptable discrimination performance with a summary c-statistic of 0.714; 95% CI 0.681–0.747 for R-FSRS and 0.709; 95% CI 0.647–0.769 for Basic IS (*Figure 4*). After excluding results from the development cohorts, both models showed reduced discriminatory performance with a summary c-statistic of 0.667; 95% CI 0.604–0.727 for R-FSRS and 0.701; 95% CI 0.583–0.807 for Basic IS (see Supplementary material online, *Figure S1*). Most studies evaluated the R-FSRS model separately in men and women. Analysis according to sex revealed similar discriminatory performance in men (summary c-statistic 0.706, 95% CI 0.657–0.755) and women (0.716; 95% CI 0.667–0.761) (see Supplementary material online, *Figure S2*).

Analysis comparing regression models with machine learning models showed better performance in machine learning models, with a summary c-statistic of 0.853; 95% CI 0.823–0.882 for machine learning models and a summary c-statistic of 0.732; 95% CI 0.718–0.745 for regression models (see Supplementary material online, *Figure S3*). Funnel plots were symmetrical but with additional horizontal scatter, consistent with the presence of between-study heterogeneity (see Supplementary material online, *Figure S4*).

## Discussion

This systematic review and meta-analysis included 80 models developed and/or validated in 48 cohorts representative of, and including variables readily available in, the general population for estimating an individual’s average risk of incident stroke. The majority of models showed good discrimination performance, but only two models (R-FSRS and Basic IS) were eligible for primary meta-analysis and demonstrated acceptable summary discrimination performance measures. However, after excluding the derivation cohorts and studies at high risk of bias, the performance of both models reduced to the point that they may not offer good discrimination of stroke. Additionally, clinical utility remains uncertain as neither of the models underwent a prospective investigation of clinical or cost-effectiveness.

### Previous work

A recent review of prediction models for incident stroke included 17 studies until February 2022.^18^ However, it included models that have been derived and/or validated in specific populations and models with variables not readily available in the general population, limiting the clinical translatability of the findings to the primary prevention setting in the community. Furthermore, the authors performed a meta-analysis of all the included models together, rather than delineating whether a particular model performed particularly well in practice.^18^

Other reviews have focused on the prediction of stroke in specific groups, not reflective of the general population, for example, in patients on dialysis.^24^ Some reviews explored composite scores for vascular disease, but these did not specifically address stroke,^21,23^ and others included scores with variables that are not usually readily available in the general population.^20,22^

### Clinical relevance

Given that the burden of stroke and related complications is rising,^2–5^ primary prevention is an increasingly important priority. Although there have been advances in reperfusion therapies in some geographies,^77^ primary prevention remains the most effective method to reduce the overall burden of stroke.^8^ At present, stroke prevention is often encompassed within general cardiovascular disease prevention strategies.^78,79^ Yet the increasing incidence of stroke contrasts with the reductions seen in myocardial infarction (MI) and heart failure (HF),^80,81^ and whilst there are common risk factors, there are some notable differences between stroke and other conditions. For instance, evidence seems to suggest atrial fibrillation is a stronger risk factor for stroke than MI,^82^ and there may be a more pronounced association between hypertension and stroke than other cardiovascular conditions.^83^ Therefore, primary prevention of stroke may require a more tailored approach, and stroke-specific risk prediction models may prove useful to delineate those at particularly high risk in general medicine and primary care settings.

Guidelines for the primary prevention of stroke emphasize the use of risk prediction tools to aid in employing effective strategies to prevent incident stroke.^15–17^ They highlight the importance of simple and widely applicable tools and the need for further validation of current tools in different groups of the population.^8^ The models included in this review utilize variables that are readily available and easily obtainable in the community. Though many models were identified within this review, there were significant shortcomings in the available evidence. First, the majority were only evaluated in the derivation cohort, and only a small number were externally validated. Second, there was a high level of potential bias and poor reporting of calibration metrics, in line with general trends of prediction model studies.^84^ Third, we did not find any evidence of clinical utility analyses or clinical impact studies, limiting any conclusions regarding the effects of prediction models when used in a clinical pathway.

This review underscores that no current model is ready for clinical use in primary prevention pathways for stroke. Whilst there is no lack of interest in developing models, we would recommend a greater emphasis on validating, updating and prospectively testing existing models across diverse populations. Incorporating routinely collected data and assessing calibration early in model design and embedding evaluation of clinical utility in studies are essential to determine which models should be considered for prospective testing. Furthermore, the effect of the implementation of models within pathways on at least the control of risk factors for stroke is essential before one can consider translating stroke prediction models to clinical practice.

### Strengths and limitations

We employed a comprehensive search strategy to identify relevant articles and models. We aimed to maximize applicability by only including models from the general population that incorporated variables readily available in such settings.

We also acknowledge the limitations of our study. Meta-analysis of calibration performance was not possible due to a lack of calibration reporting. We did not present meta-regression or subgroup meta-analysis to investigate heterogeneity between studies based on study-level characteristics or subgroups in the absence of available individual patient data, given that such analyses would be prone to ecological bias,^85^ and are inferior to subgroup results derived with patient–level data.^26^ The funnel plot demonstrated between-study heterogeneity, which may reflect differences in the characteristics, quality or population of studies, as populations varied in age, sex, comorbidities and stroke incidence. To address between-study heterogeneity, including variation in baseline stroke incidence, we used a Bayesian random-effects meta-analysis for all pooled estimates. Nonetheless, heterogeneity was considerable, and the 95% PI was large, which limits the robustness of the assessment for how the included prediction models may perform in a new dataset. Poor reporting of race and ethnicity across studies limited our ability to assess the performance of prediction models by these important sociodemographic factors, especially given the reported racial differences in stroke and other cardiovascular risk factors.^86^ Missing data was common and is a frequently observed shortfall in prediction modelling research,^87^ even in models recommended for use in healthcare.^88^ In the absence of patient-level data and model coefficients across all cohorts to quantify the relative importance of predictor variables, we were unable to assess which predictors contributed the most to prediction across all models.

## Conclusion

Many models have been derived for stroke prediction, however, they are rarely externally validated, and studies are limited by a high risk of bias, poor reporting of calibration and a lack of clinical utility analysis or prospective validation. Thus, the evidence base is insufficient to translate these models to clinical practice.

## Supplementary Material

ztaf147_Supplementary_Data

## Data Availability

The data underlying this article will be shared on reasonable request to the corresponding author.
