# Supplementary material for: Prediction models for incident stroke in the community: a systematic review and meta-analysis of predictive performance
Source: Eur Heart J Digit Health. 2026 Feb 5;7(2):ztaf147. doi: 10.1093/ehjdh/ztaf147 (PMC12893212; doi:10.1093/ehjdh/ztaf147)
Supplement: ztaf147_Supplementary_Data [file ztaf147_supplementary_data.docx]

**Supplementary Appendix**

Prediction models for incident stroke in the community: a systematic review and meta-analysis of predictive performance

[SUPPLEMENTARY METHODS 3](#_Toc198206927)

[Formulation of research question using CHARMS (CHecklist for critical Appraisal and data extraction for systematic Reviews of prediction Modelling Studies): 3](#_Toc198206928)

[Search Terms and search strategy with full results 4](#_Toc198206929)

[Inclusion and exclusion criteria pertaining to variables incorporated in models 6](#_Toc198206930)

[PROBAST: Justifications for assessments for specific signalling questions 7](#_Toc198206931)

[Excluded studies 8](#_Toc198206932)

[Bayesian meta-analysis of *c*-statistic / AUROC 10](#_Toc198206933)

[GRADE: Standards used for judging criteria for downgrading and upgrading certainty of evidence 11](#_Toc198206934)

[SUPPLEMENTARY RESULTS 12](#_Toc198206935)

[Studies included in systematic review and meta-analysis 12](#_Toc198206936)

[Supplementary Table S1: Characteristics of included studies 14](#_Toc198206937)

[Supplementary Table S2: Outcomes of studies reporting on prediction models 16](#_Toc198206938)

[Supplementary Table S3. Prediction models and outcome definitions 21](#_Toc198206939)

[Supplementary Table S4. Prediction models by type 23](#_Toc198206940)

[Supplementary Table S5: Baseline variables used in regression models 26](#_Toc198206941)

[Supplementary Table S6: Baseline variables used in machine learning models 28](#_Toc198206942)

[Supplementary Table S7: Risk of bias and applicability assessment for each PROBAST domain 29](#_Toc198206943)

[Supplementary Figure S1: Sensitivity analysis including derivation cohorts and studies with high risk of bias, separated according to model 32](#_Toc198206944)

[Supplementary Figure S2: Sensitivity analysis for prediction models after excluding studies with high risk of bias and excluding derivation cohorts 34](#_Toc198206945)

[Supplementary Figure S3: Analysis of R-FSRS according to sex 36](#_Toc198206946)

[Supplementary Figure S4: Forest plot of regression and machine learning models 37](#_Toc198206947)

[Supplementary Figure S5: Funnel plot 38](#_Toc198206948)

[PRISMA CHECKLIST 40](#_Toc198206949)

[PRISMA ABSTRACT CHECKLIST 43](#_Toc198206950)

[REFERENCES 44](#_Toc198206951)

# SUPPLEMENTARY METHODS

## Formulation of research question using CHARMS (CHecklist for critical Appraisal and data extraction for systematic Reviews of prediction Modelling Studies):

| **CHARMS key items to guide framing of review, search strategy and study inclusion and exclusion criteria** | **Comments for this systematic review** |
| --- | --- |
| Prognostic versus diagnostic prediction model | Prognostic prediction model |
| Intended scope of the review | Models to inform referral for diagnostic testing |
| Types of Prediction modelling studies | Prediction model development without external validation in independent data, prediction model development with external validation in independent data, external model validation, possibly with model updating |
| Target population to whom the prediction model applies | Adults in the general population |
| Outcome to be predicted | Specific future event, diagnosis of stroke |
| Time span of prediction | Any time interval |
| Intended moment of using the model | Models to be used in adults in the community to predict risk of development of stroke in the future, and inform targeted screening and/or primary prevention |

## Search Terms and search strategy with full results

This search was adapted from *Poorthuis et al, Ammemwerth et al and Nadarajah et al*.^1–3^

Database(s): Ovid MEDLINE(R) ALL <1946 to November 03, 2025>

Search strategy:

| # | Searches | Results |
| --- | --- | --- |
| 1 | ((prediction* or probability) adj2 stroke).tw,kw. | 1497 |
| 2 | ((predictive or prediction) adj model*).tw,kw. | 96843 |
| 3 | ((predictive or prediction) adj rule*).tw,kw. | 2738 |
| 4 | (risk adj (score* or model* or algorithm* or calculator* or assessment)).tw,kw. | 163105 |
| 5 | c-statistic*.tw. | 11568 |
| 6 | c-index*.tw. | 13962 |
| 7 | or/1-6 [prediction models] | 268780 |
| 8 | exp Stroke/ | 195312 |
| 9 | (stroke or strokes).ti. | 155673 |
| 10 | cerebrovascular accident*.ti. | 1561 |
| 11 | CVA.ti. | 187 |
| 12 | CVAs.ti. | 14 |
| 13 | or/8-12 [stroke] | 245407 |
| 14 | 7 and 13 [prediction models and stroke] | 5884 |
| 15 | letter.pt. | 1314211 |
| 16 | case reports.pt. | 2512517 |
| 17 | case report.ti. | 377876 |
| 18 | comment.pt. | 1057563 |
| 19 | editorial.pt. | 739639 |
| 20 | interview.pt. | 31649 |
| 21 | or/15-20 [excluded study types] | 4723672 |
| 22 | 14 not 21 [search strategy without excluded studies] | 5717 |
| 23 | exp animals/ not humans.sh. | 5390144 |
| 24 | 22 not 23 [human only studies] | 5695 |

Database(s): Embase Classic+Embase <1947 to November 03, 2025>

Search Strategy:

| **#** | Searches | Results |
| --- | --- | --- |
| 1 | ((prediction* or probability) adj2 stroke).tw,kw. | 2261 |
| 2 | ((predictive or prediction) adj model*).tw,kw. | 126341 |
| 3 | ((predictive or prediction) adj rule*).tw,kw. | 3899 |
| 4 | (risk adj (score* or model* or algorithm* or calculator* or assessment)).tw,kw. | 231964 |
| 5 | c-statistic*.tw. | 19419 |
| 6 | c-index*.tw. | 19250 |
| 7 | or/1-6 [prediction models] | 372188 |
| 8 | exp Stroke/ | 533176 |
| 9 | (stroke or strokes).ti. | 247168 |
| 10 | cerebrovascular accident*.ti. | 2412 |
| 11 | CVA.ti. | 378 |
| 12 | CVAs.ti. | 24 |
| 13 | or/8-12 [stroke] | 593470 |
| 14 | 7 and 13 [prediction models and stroke] | 16944 |
| 15 | (letter or comment*).ti. | 277268 |
| 16 | case report/ or case study/ | 3393934 |
| 17 | editorial.pt. | 851805 |
| 18 | letter.pt. or letter/ | 1405432 |
| 19 | note.pt. | 1023718 |
| 20 | conference abstract.pt. | 5673896 |
| 21 | or/15-20 [excluded study types] | 11624321 |
| 22 | 14 not 21 [search strategy without excluded studies] | 10558 |
| 23 | animal/ not human/ | 1675356 |
| 24 | nonhuman/ | 8362608 |
| 25 | exp animal experiment/ | 3455072 |
| 26 | exp experimental animal/ | 951664 |
| 27 | animal model/ | 1984099 |
| 28 | exp rodent/ | 4875858 |
| 29 | (rat or rats or mouse or mice).ti. | 1928677 |
| 30 | or/23-29 [excluding animal models] | 11398218 |
| 31 | 22 not 30 [human only studies] | 10389 |

## Inclusion and exclusion criteria pertaining to variables incorporated in models

In this review we were interested in models that could be used with data routinely-collected in the community to inform the individual risk of stroke. To make the model useful it should not require additional visits for baseline risk stratification.

The information that was considered likely to be available in community-based settings were:

- Sociodemographic variables including but not limited to age, sex, and ethnicity.
- Disease conditions and procedures including but not limited to hypertension, diabetes mellitus, cardiovascular disease, and atrial fibrillation.
- Clinical assessments including but not limited to heart rate, systolic and diastolic blood pressure, height, weight and body mass index.
- Medications prescribed including but not limited to antihypertensives, statins, antidepressants, anxiolytics/hypnotics and antipsychotics.
- Lifestyle factors including but not limited to smoking status and alcohol consumption.
- Simple laboratory tests and biomarkers including but not limited to total, high-density lipoprotein and low-density lipoprotein cholesterol and triglycerides.

We excluded the following types of variables that are not routinely available in community records, or are very rarely tested for in community clinical practice and so are not generalisable:

- Analysis of waist circumference, exercise and diet
- Analysis of electrocardiograph (ECG) parameters
- Analysis of results of blood tests not routinely collected, such as uric acid and homocysteine levels

## PROBAST: Justifications for assessments for specific signalling questions

Each model was assessed for risk of bias as either “high”, “unclear” or “low” in four domains (participants, predictors, outcomes and analysis) through a range of signalling questions. Applicability to our review question was assessed for each model in three domains (participants, predictors and outcomes) using the same scale.^4^

Risk of bias

Domain 4: Analysis

Signalling question 4: Were participants with missing data handled appropriately?

We assessed risk of bias for missing data by assuming that if missing data was not mentioned at all that it was likely missing but not taken account for and thus marked as ‘N or PN’ and thus ‘high’ risk of bias. If there was any extent of missing data and methods had not been used to impute or an analysis had not been made to assess whether inclusion of missing values would have made a difference to performance measures, then the signalling question was marked as ‘N or PN’ and the overall domain as ‘high’ risk of bias.

## Excluded studies

**Studies excluded that met a number of inclusion criteria but utilised data not routinely available in a community setting**

1. Wolf PA, D'Agostino RB, Belanger AJ, Kannel WB. Probability of stroke: a risk profile from the Framingham Study. Stroke. 1991 Mar;22(3):312-8
2. Wang Q, Zhang L, Li Y, Tang X, Yao Y, Fang Q. Development of stroke predictive model in community-dwelling population: A longitudinal cohort study in Southeast China. Front Aging Neurosci. 2022 Dec 22;14:1036215
3. Chun M, Clarke R, Zhu T, Clifton D, Bennett D, Chen Y, Guo Y, Pei P, Lv J, Yu C, Yang L, Li L, Chen Z, Cairns BJ; China Kadoorie Biobank Collaborative Group. Publisher Correction: Utility of single versus sequential measurements of risk factors for prediction of stroke in Chinese adults. Sci Rep. 2021 Oct 18;11(1):20874
4. Ding Z, Zhang L, Niu M, Zhao B, Liu X, Huo W, Hou J, Mao Z, Wang Z, Wang C. Stroke prevention in rural residents: development of a simplified risk assessment tool with artificial intelligence. Neurol Sci. 2023 May;44(5):1687-1694
5. Lumley T, Kronmal RA, Cushman M, Manolio TA, Goldstein S. A stroke prediction score in the elderly: validation and Web-based application. J Clin Epidemiol. 2002 Feb;55(2):129-36
6. Manuel DG, Tuna M, Perez R, Tanuseputro P, Hennessy D, Bennett C, Rosella L, Sanmartin C, van Walraven C, Tu JV. Predicting Stroke Risk Based on Health Behaviours: Development of the Stroke Population Risk Tool (SPoRT). PLoS One. 2015 Dec 4;10(12):e0143342
7. Parmar P, Krishnamurthi R, Ikram MA, Hofman A, Mirza SS, Varakin Y, Kravchenko M, Piradov M, Thrift AG, Norrving B, Wang W, Mandal DK, Barker-Collo S, Sahathevan R, Davis S, Saposnik G, Kivipelto M, Sindi S, Bornstein NM, Giroud M, Béjot Y, Brainin M, Poulton R, Narayan KM, Correia M, Freire A, Kokubo Y, Wiebers D, Mensah G, BinDhim NF, Barber PA, Pandian JD, Hankey GJ, Mehndiratta MM, Azhagammal S, Ibrahim NM, Abbott M, Rush E, Hume P, Hussein T, Bhattacharjee R, Purohit M, Feigin VL; Stroke RiskometerTM Collaboration Writing Group. The Stroke Riskometer(TM) App: validation of a data collection tool and stroke risk predictor. Int J Stroke. 2015 Feb;10(2):231-44
8. Silventoinen K, Pankow J, Lindström J, Jousilahti P, Hu G, Tuomilehto J. The validity of the Finnish Diabetes Risk Score for the prediction of the incidence of coronary heart disease and stroke, and total mortality. Eur J Cardiovasc Prev Rehabil. 2005 Oct;12(5):451-8
9. Huynh TB, McClure LA, Howard VJ, Stafford MM, Judd SE, Burstyn I. Duration of employment within occupations and incident stroke in a US general population cohort 45 years of age or older (REGARDS study). Am J Ind Med. 2023 Feb;66(2):142-154

**Studies excluded that met a number of inclusion criteria but participant inclusion was restricted to an at risk population subset (e.g. those with a diagnosis of hypertension) such that they were not representative of the general population.**

1. Singer DE, Chang Y, Borowsky LH, Fang MC, Pomernacki NK, Udaltsova N, Reynolds K, Go AS. A new risk scheme to predict ischemic stroke and other thromboembolism in atrial fibrillation: the ATRIA study stroke risk score. J Am Heart Assoc. 2013 Jun 21;2(3):e000250
2. Woo SH, Marhefka GD, Cowan SW, Ackermann L. Development and Validation of a Prediction Model for Stroke, Cardiac, and Mortality Risk After Non-Cardiac Surgery. J Am Heart Assoc. 2021 Feb 16;10(4):e018013
3. Wu X, Hu M, Zhang J, Li K, Yang X. A New Predictive Model for In-Hospital Major Adverse Cardiac and Cerebrovascular Events in Chinese Patients After Major Noncardiac Surgery. Am J Cardiol. 2023 Jan 1;186:196-202
4. Zhang X, Fei N, Zhang X, Wang Q, Fang Z. Machine Learning Prediction Models for Postoperative Stroke in Elderly Patients: Analyses of the MIMIC Database. Front Aging Neurosci. 2022 Jul 18;14:897611
5. Lip GYH, Tran G, Genaidy A, Marroquin P, Estes C, Landsheft J. Improving dynamic stroke risk prediction in non-anticoagulated patients with and without atrial fibrillation: comparing common clinical risk scores and machine learning algorithms. Eur Heart J Qual Care Clin Outcomes. 2022 Aug 17;8(5):548-556
6. Mitchell LB, Southern DA, Galbraith D, Ghali WA, Knudtson M, Wilton SB; APPROACH investigators. Prediction of stroke or TIA in patients without atrial fibrillation using CHADS2 and CHA2DS2-VASc scores. Heart. 2014 Oct;100(19):1524-30
7. Nishi H, Oishi N, Ogawa H, Natsue K, Doi K, Kawakami O, Aoki T, Fukuda S, Akao M, Tsukahara T; Fushimi AF Registry investigators.. Predicting cerebral infarction in patients with atrial fibrillation using machine learning: The Fushimi AF registry. J Cereb Blood Flow Metab. 2022 May;42(5):746-756
8. Papadopoulou A, Harding D, Slabaugh G, Marouli E, Deloukas P. Prediction of atrial fibrillation and stroke using machine learning models in UK Biobank. Heliyon. 2024 Mar 17;10(7):e28034

**Studies excluded that met a number of inclusion criteria but the models were composite scores and did not include a specific risk score the prediction of incident stroke**

1. Saeed A, Nambi V, Sun W, Virani SS, Taffet GE, Deswal A, Selvin E, Matsushita K, Wagenknecht LE, Hoogeveen R, Coresh J, de Lemos JA, Ballantyne CM. Short-Term Global Cardiovascular Disease Risk Prediction in Older Adults. J Am Coll Cardiol. 2018 Jun 5;71(22):2527-2536
2. Shan Y, Zhang Y, Zhao Y, Lu Y, Chen B, Yang L, Tan C, Bai Y, Sang Y, Liu J, Jian M, Ruan L, Zhang C, Li T. Development and validation of a cardiovascular diseases risk prediction model for Chinese males (CVDMCM). Front Cardiovasc Med. 2022 Nov 18;9:967097
3. Selmer R, Igland J, Ariansen I, Tverdal A, Njølstad I, Furu K, Tell GS, Klemsdal TO. NORRISK 2: A Norwegian risk model for acute cerebral stroke and myocardial infarction. Eur J Prev Cardiol. 2017 May;24(7):773-782
4. Sarrafzadegan N, Hassannejad R, Marateb HR, Talaei M, Sadeghi M, Roohafza HR, Masoudkabir F, Oveisgharan S, Mansourian M, Mohebian MR, Mañanas MA. PARS risk charts: A 10-year study of risk assessment for cardiovascular diseases in Eastern Mediterranean Region. PLoS One. 2017 Dec 19;12(12):e0189389
5. Stenling A, Häggström C, Norberg M, Norström F. Lifetime risk predictions for cardiovascular diseases: Competing risks analyses on a population-based cohort in Sweden. Atherosclerosis. 2020 Nov;312:90-98
6. Veronesi G, Gianfagna F, Chambless LE, Giampaoli S, Mancia G, Cesana G, Ferrario MM. Long-term prediction of major coronary or ischaemic stroke event in a low-incidence Southern European population: model development and evaluation of clinical utility. BMJ Open. 2013 Nov 12;3(11):e003630
7. Wolfson J, Vock DM, Bandyopadhyay S, Kottke T, Vazquez-Benitez G, Johnson P, Adomavicius G, O'Connor PJ. Use and Customization of Risk Scores for Predicting Cardiovascular Events Using Electronic Health Record Data. J Am Heart Assoc. 2017 Apr 24;6(4):e003670
8. Jin C, Li J, Liu F, Li X, Hui Y, Chen S, Li F, Wang G, Liang F, Lu X, Wu S, Gu D. Life's Essential 8 and 10-Year and Lifetime Risk of Atherosclerotic Cardiovascular Disease in China. Am J Prev Med. 2023 Jun;64(6):927-935
9. Chen GX, Barajas-Martínez H, Ciconte G, Wu CI, Monasky MM, Xia H, Li B, Capra JA, Guo K, Zhang ZH, Chen X, Yang B, Jiang H, Tse G, Mak CM, Aizawa Y, Gollob MH, Antzelevitch C, Wilde AAM, Pappone C, Hu D. Clinical characteristics and electrophysiologic properties of SCN5A variants in fever-induced Brugada syndrome. EBioMedicine. 2023 Jan;87:104388
10. Al-Shamsi S. Development and validation of a novel 10-year cardiovascular risk prediction nomogram for the United Arab Emirates national population. BMJ Open. 2022 Dec 29;12(12):e064502
11. Ambale-Venkatesh B, Nguyen HT, Reis JP, Wu CO, Carr JJ, Nwabuo C, et al. Lifetime vs 10-year cardiovascular disease prediction in young adults using statistical machine learning and deep learning: The CARDIA study. medRxiv. 2022 Sep 22;2022.09.22.22280254. doi: 10.1101/2022.09.22.22280254
12. Chia YC, Lim HM, Ching SM. Validation of the pooled cohort risk score in an Asian population - a retrospective cohort study. BMC Cardiovasc Disord. 2014 Nov 20;14:163
13. Kist JM, Vos RC, Mairuhu ATA, Struijs JN, van Peet PG, Vos HMM, van Os HJA, Beishuizen ED, Sijpkens YWJ, Faiq MA, Numans ME, Groenwold RHH. SCORE2 cardiovascular risk prediction models in an ethnic and socioeconomic diverse population in the Netherlands: an external validation study. EClinicalMedicine. 2023 Feb 16;57:101862

## Bayesian meta-analysis of *c*-statistic / AUROC

All Bayesian meta-analysis models assume random effects by default. Results are based on the posterior median. Prediction intervals are directly obtained from the corresponding posterior quartiles. The standard model for random effects meta-analysis assumes that the ‘true’ performance is normally distributed within and across studies.^5^ Within-study normality of performance estimates can be justified with this selection of included studies because they are all large. *Snell et al.* showed that the between-study distribution of the *c-*statistic on the original scale is not normally distributed when there is variability in the predictor effect across studies (which is likely in this selection of studies as they include different populations, and adopt slightly different definitions for predictors).^5^ They found that the logit scale is more appropriate for the estimation of prediction interval. Consequently we used the “valmeta” function of the “metamisc” package in R software which applies a logit transformation to the *c-*statistic prior to calculation of summary *c-*statistic and prediction interval.^6^

For appropriate prior distributions we borrowed from earlier work by *Debray et al.* which recommended a half Student*-t* distribution with location *m*, scale σ, and *v* degrees of freedom where we set *m* = 0 and define σ equal to the largest empirical value of
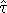
 (to allow for more extreme values of heterogeneity).^7^ These hyperparameter values allow to penalise the extent of between-study heterogeneity when the number of included validation studies is low.^7^ Further we also used *v* = 3 to ensure that the variance σ^2^ *v*/(*v*-2) exists and samples of τ were truncated above 10 to rule out unreasonable values. Thus the resulting priors are given as τdiscr ~ Student−*t*(0, 0.5^2^, 3)T[0.10] which has been shown to allow for large but realistic values for between-study heterogeneity.^7^

## GRADE: Standards used for judging criteria for downgrading and upgrading certainty of evidence

The certainty of the evidence was graded as ‘high’ (further research is very unlikely to change our confidence in the effect estimate), ‘moderate’ (further research is likely to have an important impact on our confidence in the effect estimate), ‘low’ (further research is very likely to have an important impact on our confidence in the effect estimate and is likely to change the estimate) or ‘very low’ (any estimate of effect is very uncertain).

The initial certainty level of the included prediction modelling studies was set at ‘high’ because the association between the predictors and outcomes was considered irrespective of any causal connection.^8^ Eight criteria were considered to further downgrade or upgrade the certainty of the evidence; five criteria which might downgrade the overall certainty of the evidence (methodological limitations of the study, indirectness, imprecision, inconsistency and likelihood of publication bias) and three which might potentially upgrade the overall certainty of the evidence (large effect, dose-response relation in the effect, and opposing plausible residual bias or confounding).

Methodological limitations of the studies were assessed by considering the overall risk of bias judgement across studies based on the overall PROBAST risk of bias assessment. Indirectness was assessed by making a global judgement on how dissimilar the research evidence is to the research question at hand (in terms of population and outcomes across studies).

Indirectness was assessed through concerns regarding the applicability of each included study from PROBAST (i.e. when the populations, predictors or outcomes of the study differ from the research question) and an overall judgement across studies was made.

Imprecision was assessed by considering the optimal total number of events across all studies. A minimum threshold of 10 events per variable was considered as the minimum required in regression modelling development studies, and 100 when machine learning methods had been used.^9,10^ For external validation studies a minimum sample size of at least 200 events was less concerning for imprecision.^11^ Results may also be imprecise when the 95% confidence intervals of c-statistic of all studies or of the largest studies include insufficient discrimination performance (0.5).

A global judgement on inconsistency was evaluated through the consistency of the model discrimination performance and the range of the 95% PI as a statistical measure of heterogeneity. Widely differing estimates of the c-statistic indicated inconsistency or if the 95% PI of the summary c-statistic was wide and included 0.5.

Publication bias was suspected when the body of evidence consisted of only positive studies from small sample sizes or all studies were funded by industry.

A large magnitude of effect (i.e. highly discriminatory predictive performance) was considered if the c-statistic exceeded 0.7 in the majority of studies.^12^ Since this review was not focused on drugs or pharmaceutical agents, assessing a dose-response gradient was not applicable here. Finally, we only included studies that described a multivariable prediction model and thus making a judgement whether all plausible confounders and biases were accounted for and may lead to an underestimated association is not applicable here.

# SUPPLEMENTARY RESULTS

## Studies included in systematic review and meta-analysis

1. Arafa A, Kokubo Y, Sheerah HA, Sakai Y, Watanabe E, Li J, Honda-Kohmo K, Teramoto M, Kashima R, Nakao YM, Koga M. Developing a Stroke Risk Prediction Model Using Cardiovascular Risk Factors: The Suita Study. Cerebrovasc Dis. 2022;51(3):323-330
2. Assmann G, Schulte H, Cullen P, Seedorf U. Assessing risk of myocardial infarction and stroke: new data from the Prospective Cardiovascular Münster (PROCAM) study. Eur J Clin Invest. 2007 Dec;37(12):925-32
3. Borglykke A, Andreasen AH, Kuulasmaa K, Sans S, Ducimetière P, Vanuzzo D, Ferrario MM, Palmieri L, Karvanen J, Tunstall-Pedoe H, Jørgensen T; MORGAM Project. Stroke risk estimation across nine European countries in the MORGAM project. Heart. 2010 Dec;96(24):1997-2004
4. Camen S, Palosaari T, Reinikainen J, Sprünker NA, Niiranen T, Gianfagna F, Vishram-Nielsen JKK, Costanzo S, Söderberg S, Palmieri L, Ferrario M, Peters A, Vartiainen E, Donati MB, Donfrancesco C, Borchini R, Börschel CS, Giampaoli S, Di Castelnuovo A, Magnussen C, Kee F, Koenig W, Blankenberg S, de Gaetano G, Tunstall-Pedoe H, Rospleszcz S, Jørgensen T, Zeller T, Kuulasmaa K, Linneberg A, Salomaa V, Iacoviello L, Schnabel RB; BiomarCaRE Consortium. Cardiac Troponin I and Incident Stroke in European Cohorts: Insights From the BiomarCaRE Project. Stroke. 2020 Sep;51(9):2770-2777
5. Chambless LE, Heiss G, Shahar E, Earp MJ, Toole J. Prediction of ischemic stroke risk in the Atherosclerosis Risk in Communities Study. Am J Epidemiol. 2004 Aug 1;160(3):259-69. doi: 10.1093/aje/kwh189. Erratum in: Am J Epidemiol. 2004 Nov 1;160(9):927
6. Chien KL, Su TC, Hsu HC, Chang WT, Chen PC, Sung FC, Chen MF, Lee YT. Constructing the prediction model for the risk of stroke in a Chinese population: report from a cohort study in Taiwan. Stroke. 2010 Sep;41(9):1858-64
7. Chun M, Clarke R, Zhu T, Clifton D, Bennett DA, Chen Y, Guo Y, Pei P, Lv J, Yu C, Yang L, Li L, Chen Z, Cairns BJ; China Kadoorie Biobank Collaborative Group. Development, validation and comparison of multivariable risk scores for prediction of total stroke and stroke types in Chinese adults: a prospective study of 0.5 million adults. Stroke Vasc Neurol. 2022 Aug;7(4):328-336
8. Di Castelnuovo A, Veronesi G, Costanzo S, Zeller T, Schnabel RB, de Curtis A, Salomaa V, Borchini R, Ferrario M, Giampaoli S, Kee F, Söderberg S, Niiranen T, Kuulasmaa K, de Gaetano G, Donati MB, Blankenberg S, Iacoviello L; BiomarCaRE Investigators. NT-proBNP (N-Terminal Pro-B-Type Natriuretic Peptide) and the Risk of Stroke. Stroke. 2019 Mar;50(3):610-617
9. Ferket BS, van Kempen BJ, Wieberdink RG, Steyerberg EW, Koudstaal PJ, Hofman A, Shahar E, Gottesman RF, Rosamond W, Kizer JR, Kronmal RA, Psaty BM, Longstreth WT Jr, Mosley T, Folsom AR, Hunink MG, Ikram MA. Separate prediction of intracerebral hemorrhage and ischemic stroke. Neurology. 2014 May 20;82(20):1804-12
10. Flueckiger P, Longstreth W, Herrington D, Yeboah J. Revised Framingham Stroke Risk Score, Nontraditional Risk Markers, and Incident Stroke in a Multiethnic Cohort. Stroke. 2018 Feb;49(2):363-369
11. Foraker RE, Greiner M, Sims M, Tucker KL, Towfighi A, Bidulescu A, Shoben AB, Smith S, Talegawkar S, Blackshear C, Wang W, Hardy NC, O'Brien E. Comparison of risk scores for the prediction of stroke in African Americans: Findings from the Jackson Heart Study. Am Heart J. 2016 Jul;177:25-32
12. Harada A, Ueshima H, Kinoshita Y, Miura K, Ohkubo T, Asayama K, Ohashi Y; Japan Arteriosclerosis Longitudinal Study Group. Absolute risk score for stroke, myocardial infarction, and all cardiovascular disease: Japan Arteriosclerosis Longitudinal Study. Hypertens Res. 2019 Apr;42(4):567-579
13. Hilvo M, Dhar I, Lääperi M, Lysne V, Sulo G, Tell GS, Jousilahti P, Nygård OK, Brenner H, Schöttker B, Laaksonen R. Primary cardiovascular risk prediction by LDL-cholesterol in Caucasian middle-aged and older adults: a joint analysis of three cohorts. Eur J Prev Cardiol. 2022 Mar 25;29(3):e128-e137
14. Hong C, Pencina MJ, Wojdyla DM, Hall JL, Judd SE, Cary M, Engelhard MM, Berchuck S, Xian Y, D'Agostino R Sr, Howard G, Kissela B, Henao R. Predictive Accuracy of Stroke Risk Prediction Models Across Black and White Race, Sex, and Age Groups. JAMA. 2023 Jan 24;329(4):306-317
15. Howard G, McClure LA, Moy CS, Howard VJ, Judd SE, Yuan Y, Long DL, Muntner P, Safford MM, Kleindorfer DO. Self-Reported Stroke Risk Stratification: Reasons for Geographic and Racial Differences in Stroke Study. Stroke. 2017 Jul;48(7):1737-1743
16. Hung CY, Lin CH, Lan TH, Peng GS, Lee CC. Development of an intelligent decision support system for ischemic stroke risk assessment in a population-based electronic health record database. PLoS One. 2019 Mar 13;14(3):e0213007
17. Hung CY, Lin CH and Lee CC. Improving Young Stroke Prediction by Learning with Active Data Augmenter in a Large-Scale Electronic Medical Claims Database. 2018 40th Annual International Conference of the IEEE Engineering in Medicine and Biology Society (EMBC), Honolulu, HI, USA, 2018, pp. 5362-5365
18. Hunter E, Kelleher JD. Age Specific Models to Capture the Change in Risk Factor Contribution by Age to Short Term Primary Ischemic Stroke Risk. Front Neurol. 2022 Feb 17;13:803749
19. Jung KJ, Hwang S, Lee S, Kim HC, Jee SH. Traditional and Genetic Risk Score and Stroke Risk Prediction in Korea. Korean Circ J. 2018 Aug;48(8):731-740
20. Lee S, Lee H, Kim HS, Koh SB. Incidence, Risk Factors, and Prediction of Myocardial Infarction and Stroke in Farmers: A Korean Nationwide Population-based Study. J Prev Med Public Health. 2020 Sep;53(5):313-322
21. Li X, Wang Y, Xu J. Development of a machine learning-based risk prediction model for cerebral infarction and comparison with nomogram model. J Affect Disord. 2022 Oct 1;314:341-348
22. Li, Y, M Mamouei, G Salimi-Khorshidi, S Rao, A Hassaine, D Canoy, T Lukasiewicz, and K Rahimi. 2022. “Hi-BEHRT: Hierarchical Transformer-Based Model for Accurate Prediction of Clinical Events Using Multimodal Longitudinal Electronic Health Records.” IEEE Journal of Biomedical and Health Informatics 27 (2): 1106–17
23. Lolak S, Attia J, McKay GJ, Thakkinstian A. Comparing Explainable Machine Learning Approaches With Traditional Statistical Methods for Evaluating Stroke Risk Models: Retrospective Cohort Study. JMIR Cardio. 2023 Jul 26;7:e47736
24. Majed B, Tafflet M, Kee F, Haas B, Ferrieres J, Montaye M, Ruidavets JB, Arveiler D, Yarnell J, Amouyel P, Ducimetiere P, Empana JP; PRIME study group. External validation of the 2008 Framingham cardiovascular risk equation for CHD and stroke events in a European population of middle-aged men. The PRIME study. Prev Med. 2013 Jul;57(1):49-54
25. Marrugat J, Subirana I, Ramos R, Vila J, Marín-Ibañez A, Guembe MJ, Rigo F, Tormo Díaz MJ, Moreno-Iribas C, Cabré JJ, Segura A, Baena-Díez JM, de la Cámara AG, Lapetra J, Grau M, Quesada M, Medrano MJ, González Diego P, Frontera G, Gavrila D, Aicua EA, Basora J, García JM, García-Lareo M, Gutierrez JA, Mayoral E, Sala J, D'Agostino R, Elosua R; FRESCO Investigators. Derivation and validation of a set of 10-year cardiovascular risk predictive functions in Spain: the FRESCO Study. Prev Med. 2014 Apr;61:66-74
26. Teoh D. Towards stroke prediction using electronic health records. BMC Med Inform Decis Mak. 2018 Dec 4;18(1):127
27. Wannamethee SG, Shaper AG, Lennon L, Morris RW. Metabolic syndrome vs Framingham Risk Score for prediction of coronary heart disease, stroke, and type 2 diabetes mellitus. Arch Intern Med. 2005 Dec 12-26;165(22):2644-50
28. Wu Y, Fang Y. Stroke Prediction with Machine Learning Methods among Older Chinese. Int J Environ Res Public Health. 2020 Mar 12;17(6):1828
29. Yang K, Chen M, Wang Y, Jiang G, Hou N, Wang L, Wen K, Li W. Development of a predictive risk stratification tool to identify the population over age 45 at risk for new-onset stroke within 7 years. Front Aging Neurosci. 2023 Jun 14;15:1101867
30. Yatsuya H, Iso H, Li Y, Yamagishi K, Kokubo Y, Saito I, Sawada N, Inoue M, Tsugane S. Development of a Risk Equation for the Incidence of Coronary Artery Disease and Ischemic Stroke for Middle-Aged Japanese　- Japan Public Health Center-Based Prospective Study. Circ J. 2016 May 25;80(6):1386-95
31. Yatsuya H, Iso H, Yamagishi K, Kokubo Y, Saito I, Suzuki K, Sawada N, Inoue M, Tsugane S. Development of a point-based prediction model for the incidence of total stroke: Japan public health center study. Stroke. 2013 May;44(5):1295-302
32. Zhang XF, Attia J, D'Este C, Yu XH, Wu XG. A risk score predicted coronary heart disease and stroke in a Chinese cohort. J Clin Epidemiol. 2005 Sep;58(9):951-8
33. Zhang Y, Fang X, Guan S, Wu X, Liu H, Wang C, Zhang Z, Gu X, Liu C, Cheng J. Validation of 10-Year Stroke Prediction Scores in a Community-Based Cohort of Chinese Older Adults. Front Neurol. 2020 Oct 22;11:986
34. Dufouil C, Beiser A, McLure LA, Wolf PA, Tzourio C, Howard VJ, Westwood AJ, Himali JJ, Sullivan L, Aparicio HJ, Kelly-Hayes M, Ritchie K, Kase CS, Pikula A, Romero JR, D'Agostino RB, Samieri C, Vasan RS, Chêne G, Howard G, Seshadri S. Revised Framingham Stroke Risk Profile to Reflect Temporal Trends. Circulation. 2017 Mar 21;135(12):1145-1159
35. Hung CY, Chen WC, Lai PT, Lin CH, Lee CC. Comparing deep neural network and other machine learning algorithms for stroke prediction in a large-scale population-based electronic medical claims database. Annu Int Conf IEEE Eng Med Biol Soc. 2017 Jul;2017:3110-3113
36. Sun L, Pennells L, Kaptoge S, Nelson CP, Ritchie SC, Abraham G, Arnold M, Bell S, Bolton T, Burgess S, Dudbridge F, Guo Q, Sofianopoulou E, Stevens D, Thompson JR, Butterworth AS, Wood A, Danesh J, Samani NJ, Inouye M, Di Angelantonio E. Polygenic risk scores in cardiovascular risk prediction: A cohort study and modelling analyses. PLoS Med. 2021 Jan 14;18(1):e1003498
37. Xing X, Yang X, Liu F, Li J, Chen J, Liu X, Cao J, Shen C, Yu L, Lu F, Wu X, Zhao L, Li Y, Hu D, Lu X, Gu D. Predicting 10-Year and Lifetime Stroke Risk in Chinese Population. Stroke. 2019 Sep;50(9):2371-2378
38. Bos D, Ikram MA, Leening MJG, Ikram MK. The Revised Framingham Stroke Risk Profile in a Primary Prevention Population: The Rotterdam Study. Circulation. 2017 May 30;135(22):2207-2209
39. D'Agostino RB Sr, Vasan RS, Pencina MJ, Wolf PA, Cobain M, Massaro JM, Kannel WB. General cardiovascular risk profile for use in primary care: the Framingham Heart Study. Circulation. 2008 Feb 12;117(6):743-53
40. Li Y, Salimi-Khorshidi G, Rao S, Canoy D, Hassaine A, Lukasiewicz T, Rahimi K, Mamouei M. Validation of risk prediction models applied to longitudinal electronic health record data for the prediction of major cardiovascular events in the presence of data shifts. Eur Heart J Digit Health. 2022 Oct 21;3(4):535-547

## Supplementary Table S1: Characteristics of included studies

| **Study** | **Study aim** | **Age**  **(mean ±SD)** | **Female (%)** | **BMI**  **(mean ±SD)** | **Diabetes (%)** | **Hypertension (%)** | **MI/IHD (%)** | **Hyperlipidaemia (%)** | **Smoking (%)** | **AF (%)** |
| --- | --- | --- | --- | --- | --- | --- | --- | --- | --- | --- |
| Arafa *et al* 2021 | D, IV | 54.7±7 | 53.8 | - | 4.9 | 22.8 | - | 17.4 | 29.3 | 1.3 |
| Assman *et al* 2007 | D | 45.7±6.8 | 27.4 | - | 6.2 | - | - | - | 29.9 | - |
| Borglykke *et al* 2010 | D, IV | - | - | - | - | - | - | - | - | - |
| Camen *et al* 2020 | EV | *50.7 (18) | 50.3 | 26.2±5.7 | 3.9 | 14.8 | - | - | 27 | - |
| Chambless *et al* 2004 | D | - | 55.2 | - | - | - | - | - | - | - |
| Chien *et al* 2010 | D, IV, EV | - | - | - | - | - | - | - | - | - |
| Chun *et al* 2022 | D, EV | 51.9±10.6 | 59.3 | - | 4.8 men,  5.3 women | - | - | - | 68.5 men,  3.1 women | - |
| Di Castelnuovo *et al* 2019 | D | 52±13 | 49.7 | 27.3±4.7 | 5 | 19.2 | 2.7 | - | 24.7 | - |
| Ferket *et al* 2014 | D | *ARIC 54 (49-59)  Rotterdam 68 (62-76)  CHS 71 (68-76) | 57.4 | *ARIC 26.9 (24,30.4), Rotterdam 26 (23.8, 28.4)  CHS 26.1 (23.5,29.2) | 11.9 | 30.4 | 13.6 | - | 22.4 | - |
| Flueckiger *et al* 2018 | EV | 62.2±10.2 | 52.8 | 28.3±5.5 | 12.7 | - | - | - | 13 | - |
| Foraker *et al* 2016 | EV | *54.5 (44.8-64.1) | 64.8 | *30.5 (26.8-35.5) | 18 | 48.3 | - | - | 12.4 | - |
| Harada *et al* 2018 | D | 61.5 | 60.4 | - | - | - | - | - | - | - |
| Hilvo *et al* 2022 | D | *48 (37-58) | 54 | 26.2 (23.5-29.3) | 5 | - | - | - | 26 | - |
| Hong *et al* 2023 | EV | 61 | 54 | - | 17.3 | 39.3 | 13.7 | 22.4 | 17.2 | 3.9 |
| Howard *et al* 2017 | D, EV | *64 (9) | 47 | - | 21 | 55 | 7 | - | 14 | 17 |
| Hung *et al* 2019 | D, IV | 35.5±20.2 | 51.5 | - | 5.8 | 11.9 | 4.8 | 7.6 | - | 0.3 |
| Hung *et al*  2018 | D, IV | - | - | - | - | - | - | - | - | - |
| Hunter *et al* 2022 | D, IV | - | 56 | 26.4 | - | - | - | - | - | - |
| Jung *et al* 2018 | D, IV | - | - | - | - | - | - | - | - | - |
| Lee *et al* 2020 | D, IV | - | 30.9 | - | - | - | - | - | - | - |
| Li X *et al* 2022 | D, IV | 63 | 31 stroke  45 no stroke | - | 11 | 48.9 | - | - | - | - |
| Li Y *et al* 2022 (1) | D, IV | 52.6±18.1 | - | - | - | - | - | - | - | - |
| Lolak *et al* 2023 | D, IV | - | 62.8 | - | 35.9 | 76.8 | - | 82.9 | - | 70.2 |
| Majed *et al* 2013 | D, IV | 54.9±2.9 | - | - | 2.3 | 13.1 | - | - | 27.6 | - |
| Marrugat *et al* 2014 | D, EV | - | 53.8 | - | - | - | - | - | - | - |
| Teoh *et al* 2018 | D, IV | - | - | - | - | - | - | - | - | - |
| Wannamethee *et al* 2005 | D, IV | 50.3±5.7 | 0 | 35.4±3.2 | 0 | 78.5 | - | 30 | 42.1 | - |
| Wu *et al* 2020 | D, IV | - | 30.4 | - | 2.3 | 25.5 | 6.9 | - | 20.8 | - |
| Yang *et al* 2023 | D, IV | 58.4±8.6 | 54 | - | 5.5 | 21.6 | 10.6 | 10 | 38.8 | - |
| Yatsuya *et al* 2016 | D, IV, EV | JPHC I 50.2±5.8  JPHC II 57.4±8.2 | JPHC I 63  JPHC II 66.1 | - | JPHC I 2.2  JPHC II 4.6 | JPHC I 10.1  JPHC II 18.3 | - | - | JPHC I 20  JPHC II 15.4 | - |
| Yatsuya et al 2013 | D, IV, EV | - | 66.1 | - | 4.6 | 18.3 | - | - | 15.4 | - |
| Zhang X *et al* 2005 | D, IV | 45±8 | 0 | 23±2.7 | - | - | - | - | 0.7 | - |
| Zhang Y *et al* 2020 | D, EV | 68.6±7.6 | 55.4 | - | 16.1 | 38.6 | - | - | 24.6 | - |
| Dufouil *et al* 2017 | D, EV | - | 65.1 | - | - | - | - | - | - | - |
| Hung *et al* 2017 | D, IV | - | - | - | - | - | - | - | - | - |
| Sun *et al* 2021 | D, IV | 56±8 | 57 | - | 1.2 | 11.2 | - | - | 10.3 | - |
| Xing *et al* 2019 | EV | MUCA 46.6±7.4  CIMIC 54.4±10.2 | 60.6 | - | MUCA 3.2  CIMIC 6.5 | - | - | - | MUCA 36.5  CIMIC 20.1 | - |
| Bos *et al* 2017 | EV | 69.5 | 58.9 | - | 13.5 | 41.8 | - | - | 18.8 | 5.3 |
| D’Agostino *et al* 2008 | D, IV, EV | 49 | 53.3 | - | 5 | 11 | - | - | 34.7 | - |
| Li Y *et al* 2022 (2) | D, IV, EV | 58±15.3 | 51.2 | 27.6±3.2 | 3.6 | - | - | - | - | 3.1 |
| Vu *et al* 2024 | D | *56 (44-65) | 54.3 | 22.5±3 | 12.2 | 31.1 | - | - | 29.5 | - |

*Data presented in median (±SD)

## Supplementary Table S2: Outcomes of studies reporting on prediction models

| **Author (cohort)** | **Model** | **Aim** | **Total** | **Events (%)** | **Discrimination** | | | **ROB participants** | **ROB**  **overall** |
| --- | --- | --- | --- | --- | --- | --- | --- | --- | --- |
|  |  |  |  |  | ***c*-statistic** | **95% CI upper** | **95% CI lower** |  |  |
| Arafa (Suita) | Suita | D/IV | 6641 | 372 (5.6) | 0.76 | 0.734 | 0.784 |  |  |
| Assman (PROCAM) | PROCAM-stroke | D | 8130 | 85 (1.05) | 0.78 | 0.727 | 0.825 |  |  |
| Borglykke (MORGAM) | Broglykke | D/IV | 88290 | 2928 (3.32) | 0.791 | 0.783 | 0.799 |  |  |
| Camen (MORGAM) | Framingham | EV | 82881 | 3033 (3.66) | 0.809 | 0.801 | 0.817 |  |  |
| Chambless (ARIC men) | Chambless | D | 6572 | 249 (3.79) | 0.744 | 0.720 | 0.767 |  |  |
| Chambless (ARIC women) | Chambless | D | 8113 | 185 (2.28) | 0.791 | 0.769 | 0.812 |  |  |
| Chien (Chin-Shan) | Clinical | D/IV | 3513 | 240 (6.83) | 0.772 | 0.744 | 0.799 |  |  |
| Chien (Chin-Shan) | Biochemical | D/IV | 3513 | 240 (6.83) | 0.773 | 0.746 | 0.8 |  |  |
| Chien (Chin-Shan) | Clinial (co-efficient based) | D/IV | 3513 | 240 (6.83) | 0.778 | 0.751 | 0.804 |  |  |
| Chien (Chin-Shan) | Biochemical (co-efficient based) | D/IV | 3513 | 240 (6.83) | 0.779 | 0.752 | 0.806 |  |  |
| Chien (Chin-Shan) | PROCAM-stroke | EV | 3513 | 240 (6.83) | 0.748 | 0.722 | 0.777 |  |  |
| Chun (CKB men) | R-FSRS | EV | 205293 | 19587 (9.54) | 0.78 | 0.77 | 0.79 |  |  |
| Chun (CKB women) | R-FSRS | EV | 298549 | 23647 (7.92) | 0.77 | 0.76 | 0.78 |  |  |
| Chun (CKB men) | R-FSRS recalibrated | D | 205293 | 19587 (9.54) | 0.78 | 0.77 | 0.79 |  |  |
| Chun (CKB women) | R-FSRS recalibrated | D | 298549 | 23647 (7.92) | 0.77 | 0.76 | 0.78 |  |  |
| Chun (CKB men) | R-FSRS refitted | D | 205293 | 19587 (9.54) | 0.79 | 0.79 | 0.8 |  |  |
| Chun (CKB women) | R-FSRS refitted | D | 298549 | 23647 (7.92) | 0.78 | 0.77 | 0.78 |  |  |
| Chun (CKB men) | R-FSRS recalibrated by geographical area | D | 205293 | 19587 (9.54) | 0.82 | 0.82 | 0.83 |  |  |
| Chun (CKB women) | R-FSRS recalibrated by geographical area | D | 298549 | 23647 (7.92) | 0.82 | 0.82 | 0.83 |  |  |
| Di Castelnuovo (BiomarCaRE) | Reference model | D | 58173 | 1550 (2.66) | 0.842 | 0.832 | 0.852 |  |  |
| Ferket (ARIC) | Basic IS | D | 15170 | 920 (6.07) | 0.789 | 0.768 | 0.811 |  |  |
| Ferket (Rotterdam) | Basic IS | D | 6910 | 820 (11.87) | 0.696 | 0.677 | 0.716 |  |  |
| Ferket (CHS) | Basic IS | D | 5413 | 819 (15.13) | 0.658 | 0.637 | 0.679 |  |  |
| Ferket (ARIC) | Basic IS | EV | 15170 | 920 (6.07) | 0.76 | 0.737 | 0.783 |  |  |
| Ferket (Rotterdam) | Basic IS | EV | 6910 | 820 (11.87) | 0.694 | 0.674 | 0.713 |  |  |
| Ferket (CHS) | Basic IS | EV | 5413 | 819 (15.13) | 0.651 | 0.63 | 0.672 |  |  |
| Ferket (ARIC) | Basic - any stroke | D | 15170 | 920 (6.07) | 0.788 | 0.767 | 0.809 |  |  |
| Ferket (Rotterdam) | Basic - any stroke | D | 6910 | 820 (11.87) | 0.69 | 0.671 | 0.709 |  |  |
| Ferket (CHS) | Basic - any stroke | D | 5413 | 819 (15.13) | 0.659 | 0.638 | 0.679 |  |  |
| Flueckiger (MESA) | R-FSRS | EV | 6712 | 231 (3.44) | 0.716 | 0.681 | 0.748 |  |  |
| Flueckiger (MESA) | PCE | EV | 6712 | 231 (3.44) | 0.716 | 0.681 | 0.748 |  |  |
| Foraker (JHS) | CVD score | EV | 4140 | 112 (2.70) | 0.79 | 0.76 | 0.83 |  |  |
| Harada (JALS) | Harada - no AF | D | 67969 | 1351 (1.99) | 0.764 | 0.751 | 0.777 |  |  |
| Harada (JALS) | Harada - AF | D | 67969 | 1351 (1.99) | 0.773 | 0.76 | 0.785 |  |  |
| Hilvo (FINRISK 2002) | Basic ACVD model | D | 7810 | 249 (3.19) | 0.817 | 0.789 | 0.842 |  |  |
| Hong (FoS, ARIC, MESA, REGARDS) | Pooled cohort equation | EV | 62482 | 2199 (3.52) | 0.72 | 0.71 | 0.73 |  |  |
| Hong (FoS, ARIC, MESA, REGARDS) | R-FSRS | EV | 62482 | 2199 (3.52) | 0.72 | 0.72 | 0.73 |  |  |
| Howard (REGARDS) | R-FSRS | EV | 23983 | 939 (3.92) | 0.71 | 0.69 | 0.73 |  |  |
| Howard (REGARDS) | SRSRF | D | 23983 | 939 (3.92) | 0.73 | 0.71 | 0.75 |  |  |
| Hung 2019 (NHIRD) | DNN | IV | 84342 | 239 (0.28) | 0.92 | 0.908 | 0.932 |  |  |
| Hung 2019 (NHIRD) | DNN | IV | 83931 | 245 (0.29) | 0.93 | 0.914 | 0.937 |  |  |
| Hung 2018 (NHIRD) | MGL | D/IV | 552898 | 4795 (0.87) | 0.763 | 0.756 | 0.770 |  |  |
| Hung 2018 (NHIRD) | SDA | D/IV | 552898 | 4795 (0.87) | 0.786 | 0.779 | 0.792 |  |  |
| Hung 2018 (NHIRD) | ADA-L | D/IV | 552898 | 4795 (0.87) | 0.797 | 0.791 | 0.803 |  |  |
| Hung 2018 (NHIRD) | ADA-M | D/IV | 552898 | 4795 (0.87) | 0.8 | 0.794 | 0.806 |  |  |
| Hung 2018 (NHIRD) | ADA-S | D/IV | 552898 | 4795 (0.87) | 0.805 | 0.799 | 0.811 |  |  |
| Hunter (FHS, FoS, FHS-3rd gen, FHS-OMNI2, FHS new offspring) | Model (all ages) | D/IV | 113714 | 2114 (1.86) | 0.69 | 0.66 | 0.72 |  |  |
| Hunter (FHS, FoS, FHS-3rd gen, FHS-OMNI2, FHS new offspring) | Model <50 | D/IV | 36815 | 106 (0.29) | 0.67 | 0.51 | 0.83 |  |  |
| Hunter (FHS, FoS, FHS-3rd gen, FHS-OMNI2, FHS new offspring) | Model- 50-59 | D/IV | 28787 | 277 (0.96) | 0.7 | 0.65 | 0.75 |  |  |
| Hunter (FHS, FoS, FHS-3rd gen, FHS-OMNI2, FHS new offspring) | Model-60-69 | D/IV | 25126 | 586 (2.33) | 0.72 | 0.69 | 0.75 |  |  |
| Hunter (FHS, FoS, FHS-3rd gen, FHS-OMNI2, FHS new offspring) | Model-70+ | D/IV | 22986 | 1145 (4.98) | 0.7 | 0.67 | 0.73 |  |  |
| Jung 2018 (KCPS-II) | TRS (Model 1) | D/IV | 144594 | 823 (0.57) | 0.58 | 0.560 | 0.599 |  |  |
| Lee S (NHIS, NHIS-NSC) | 5-year model | D/IV | 973055 | 3484 (0.36) | 0.76 | 0.752 | 0.768 |  |  |
| Li X 2022 (Jilin University Cohorts 1-3) | XgBoost | D/IV | 32366 | 15833 (48.92) | 0.91 | 0.91 | 0.92 |  |  |
| Li X 2022 (Jilin University Cohorts 1-3) | LR | D/IV | 32366 | 15833 (48.92) | 0.83 | 0.82 | 0.84 |  |  |
| Li X 2022 (Jilin University Cohorts 1-3) | SVM | D/IV | 32366 | 15833 (48.92) | 0.84 | 0.83 | 0.85 |  |  |
| Li X 2022 (Jilin University Cohorts 1-3) | RF | D/IV | 32366 | 15833 (48.92) | 0.88 | 0.88 | 0.89 |  |  |
| Li Y 2022 (CPRD) | BEHRT | D/IV | 1730828 | 224442 (12.97) | 0.89 | 0.889 | 0.891 |  |  |
| Li Y 2022 (CPRD) | Hi-BEHRT | D/IV | 1730828 | 224442 (12.97) | 0.9 | 0.899 | 0.901 |  |  |
|  |  |  |  |  |  |  |  |  |  |
| Lolak (Ramathibodi Hospital) | LR | D/IV | 275247 | 9659 (3.51) | 0.8 | 0.79 | 0.81 |  |  |
| Lolak (Ramathibodi Hospital) | BN | D/IV | 275247 | 9659 (3.51) | 0.8 | 0.79 | 0.81 |  |  |
| Lolak (Ramathibodi Hospital) | TAN | D/IV | 275247 | 9659 (3.51) | 0.83 | 0.82 | 0.83 |  |  |
| Lolak (Ramathibodi Hospital) | XGBoost | D/IV | 275247 | 9659 (3.51) | 0.89 | 0.88 | 0.89 |  |  |
| Lolak (Ramathibodi Hospital) | EBM | D/IV | 275247 | 9659 (3.51) | 0.87 | 0.86 | 0.87 |  |  |
| Majed (PRIME-combined, PRIME-France, PRIME-Ireland, Framingham) | PRIME (local) | D/IV | 9638 | 138 (1.43) | 0.68 | 0.634 | 0.723 |  |  |
| Majed (PRIME-combined, PRIME-France, PRIME-Ireland, Framingham) | Framingham | EV | 9638 | 138 (1.43) | 0.66 | 0.613 | 0.704 |  |  |
| Majed (PRIME-combined, PRIME-France, PRIME-Ireland, Framingham) | Framingham (calibrated) | EV | 9638 | 138 (1.43) | 0.66 | 0.613 | 0.704 |  |  |
| Marrugat (CORSAID, DRECA-2, MURCIA, EMMA, REGICOR, REUS, ZONA FRANCA, NAVARA, RIVANA, TALAVERA, ZACARIS) | Model A (men) | D | 13248 | 258 (1.95) | 0.714 | 0.685 | 0.743 |  |  |
| Marrugat (CORSAID, DRECA-2, MURCIA, EMMA, REGICOR, REUS, ZONA FRANCA, NAVARA, RIVANA, TALAVERA, ZACARIS) | Model A (women) | D | 15307 | 184 (1.20) | 0.747 | 0.715 | 0.779 |  |  |
| Marrugat (CORSAID, DRECA-2, MURCIA, EMMA, REGICOR, REUS, ZONA FRANCA, NAVARA, RIVANA, TALAVERA, ZACARIS) | Model A (men) | EV | 7956 | 170 (2.14) | 0.72 | 0.685 | 0.754 |  |  |
| Marrugat (CORSAID, DRECA-2, MURCIA, EMMA, REGICOR, REUS, ZONA FRANCA, NAVARA, RIVANA, TALAVERA, ZACARIS) | Model A (women) | EV | 9482 | 120 (1.27) | 0.769 | 0.727 | 0.812 |  |  |
| Marrugat (CORSAID, DRECA-2, MURCIA, EMMA, REGICOR, REUS, ZONA FRANCA, NAVARA, RIVANA, TALAVERA, ZACARIS) | Model B (men) | D | 12085 | 235 (1.95) | 0.739 | 0.709 | 0.769 |  |  |
| Marrugat (CORSAID, DRECA-2, MURCIA, EMMA, REGICOR, REUS, ZONA FRANCA, NAVARA, RIVANA, TALAVERA, ZACARIS) | Model B (women) | D | 13934 | 168 (1.21) | 0.752 | 0.718 | 0.785 |  |  |
| Marrugat (CORSAID, DRECA-2, MURCIA, EMMA, REGICOR, REUS, ZONA FRANCA, NAVARA, RIVANA, TALAVERA, ZACARIS) | Model B (men) | EV | 7956 | 170 (2.14) | 0.731 | 0.695 | 0.766 |  |  |
| Marrugat (CORSAID, DRECA-2, MURCIA, EMMA, REGICOR, REUS, ZONA FRANCA, NAVARA, RIVANA, TALAVERA, ZACARIS) | Model B (women) | EV | 9482 | 120 (1.27) | 0.781 | 0.74 | 0.821 |  |  |
| Teoh (Tsuyama Jifukai Tsuyama Chuo Hospital) | Model 1 GRU | D | 8175 | 2725 (33.33) | 0.553 | 0.547 | 0.573 |  |  |
| Teoh (Tsuyama Jifukai Tsuyama Chuo Hospital) | Model 2 CNN/RNN | D | 8175 | 2725 (33.33) | 0.56 | 0.547 | 0.573 |  |  |
| Teoh (Tsuyama Jifukai Tsuyama Chuo Hospital) | Model 3 Dual | D | 8175 | 2725 (33.33) | 0.669 | 0.658 | 0.682 |  |  |
| Wannamethee (BRHS) | MetS (10 years) | EV | 5128 | 291 (5.7) | 0.54 | 0.48 | 0.6 |  |  |
| Wannamethee (BRHS) | MetS (20 years) | EV | 5128 | 291 (5.7) | 0.55 | 0.51 | 0.59 |  |  |
| Yatsuya 2016 (JPHC II) | Final model | D/IV | 15672 | 552 (3.52) | 0.78 | 0.77 | 0.8 |  |  |
| Yatsuya 2016 (JPHC II) | Final model | EV | 11598 | 311 (2.68) | 0.76 | 0.732 | 0.786 |  |  |
| Yatsuya 2013 (JPHC I, JPHC II) | Cox model | D | 15672 | 790 (5.04) | 0.74 | 0.722 | 0.757 |  |  |
| Yatsuya 2013 (JPHC I, JPHC II) | Point-based model | D | 15672 | 790 (5.04) | 0.73 | 0.72 | 0.75 |  |  |
| Zhang X 2005 (Beijing steelworker cohort) | IS | D | 3000 | 82 (2.73) | 0.72 | 0.661 | 0.772 |  |  |
| Zhang X 2005 (Beijing steelworker cohort) | IS | IV | 1400 | 36 (2.57) | 0.78 | 0.694 | 0.847 |  |  |
| Zhang, Y 2020 (BLSA men) | R-FSRS | EV | 537 | 50 (9.31) | 0.568 | 0.524 | 0.61 |  |  |
| Zhang, Y 2020 (BLSA women) | R-FSRS | EV | 666 | 56 (8.41) | 0.575 | 0.537 | 0.613 |  |  |
| Zhang, Y 2020 (BLSA men) | Recalibrated R-FSRS | D | 537 | 50 (9.31) | 0.648 | 0.606 | 0.689 |  |  |
| Zhang, Y 2020 (BLSA women) | Recalibrated R-FSRS | D | 666 | 56 (8.41) | 0.621 | 0.583 | 0.658 |  |  |
| Zhang, Y 2020 (BLSA men) | Adjusted R-FSRS | D | 537 | 50 (9.31) | 0.568 | 0.524 | 0.61 |  |  |
| Zhang, Y 2020 (BLSA women) | Adjusted R-FSRS | D | 666 | 56 (8.41) | 0.575 | 0.537 | 0.613 |  |  |
| Dufouil (FHS men) | R-FSRS | D | 2291 | 118 (5.15) | 0.74 | 0.7 | 0.78 |  |  |
| Dufouil (FHS women) | R-FSRS | D | 2781 | 129 (4.64) | 0.78 | 0.75 | 0.82 |  |  |
| Dufouil (REGARDS men) | R-FSRS | EV | 10808 | 253 (2.34) | 0.66 | 0.63 | 0.69 |  |  |
| Dufouil (REGARDS women) | R-FSRS | EV | 12883 | 267 (2.07) | 0.71 | 0.68 | 0.74 |  |  |
| Dufouil (3C men) | R-FSRS | EV | 2949 | 95 (3.22) | 0.7 | 0.65 | 0.75 |  |  |
| Dufouil (3C women) | R-FSRS | EV | 4652 | 112 (2.41) | 0.72 | 0.68 | 0.76 |  |  |
| Hung 2017 (NHIRD) | DNN | D/IV | 798611 | 4944 (0.6) | 0.92 | 0.9 | 0.93 |  |  |
| Hung 2017 (NHIRD) | GBDT | D/IV | 798611 | 4944 (0.6) | 0.92 | 0.9 | 0.93 |  |  |
| Sun 2021 (UKB, CPRD) | Conventional risk factors (men) | D/IV | 306654 | 2347 (0.77) | 0.7 | 0.69 | 0.71 |  |  |
| Sun 2021 (UKB, CPRD) | Conventional risk factors (women) | D/IV | 306654 | 2347 (0.77) | 0.73 | 0.71 | 0.74 |  |  |
| Sun 2021 (UKB, CPRD) | Conventional risk factors + CRP (men) | D/IV | 306654 | 2347 (0.77) | 0.7 | 0.69 | 0.71 |  |  |
| Sun 2021 (UKB, CPRD) | Conventional risk factors + CRP (women) | D/IV | 306654 | 2347 (0.77) | 0.73 | 0.71 | 0.74 |  |  |
| Xing 2019 (China MUCA men) | R-FSRS | EV | 3802 | 251 (6.60) | 0.693 | 0.659 | 0.727 |  |  |
| Xing 2019 (China MUCA women) | R-FSRS | EV | 3996 | 181 (4.53) | 0.692 | 0.652 | 0.732 |  |  |
| Xing 2019 (CIMIC men) | R-FSRS | EV | 14119 | 505 (3.58) | 0.668 | 0.645 | 0.691 |  |  |
| Xing 2019 (CIMIC women) | R-FSRS | EV | 20238 | 442 (2.18) | 0.686 | 0.663 | 0.71 |  |  |
| Bos 2017 (Rotterdam men) | R-FSRS | EV | 3273 | 217 (6.63) | 0.68 | 0.65 | 0.72 |  |  |
| Bos 2017 (Rotterdam women) | R-FSRS | EV | 4693 | 285 (6.07) | 0.74 | 0.71 | 0.76 |  |  |
| d'Agostino (FHS, FOS men) | Framingham 1998 | EV | 8491 | 177 (2.08) | 0.73 | 0.71 | 0.75 |  |  |
| d'Agostino (FHS, FOS women) | Framingham 1998 | EV | 8491 | 177 (2.08) | 0.79 | 0.76 | 0.81 |  |  |
| d'Agostino (FHS, FOS) | Own model (men) | D/IV | 8491 | 177 (2.08) | 0.74 | 0.71 | 0.76 |  |  |
| d'Agostino (FHS, FOS) | Own model (women) | D/IV | 8491 | 177 (2.08) | 0.79 | 0.76 | 0.82 |  |  |
| Li 2022 (CPRD) | BEHRT | IV | 838961 | 57049 (6.80) | 0.957 | 0.955 | 0.959 |  |  |
| Li 2022 (CPRD) | BEHRT | EV | 257314 | 17497 (6.80) | 0.932 | 0.930 | 0.934 |  |  |
| Li 2022 (CPRD) | Framingham | IV | 838961 | 57049 (6.80) | 0.869 | 0.864 | 0.874 |  |  |
| Li 2022 (CPRD) | Framingham | EV | 257314 | 17497 (6.80) | 0.862 | 0.859 | 0.865 |  |  |
| Li 2022 (CPRD) | RF (QRISK) | IV | 838961 | 57049 (6.80) | 0.877 | 0.872 | 0.882 |  |  |
| Li 2022 (CPRD) | RF (QRISK) | EV | 257314 | 17497 (6.80) | 0.866 | 0.863 | 0.869 |  |  |
| Li 2022 (CPRD) | RF (Framingham) | IV | 838961 | 57049 (6.80) | 0.868 | 0.864 | 0.872 |  |  |
| Li 2022 (CPRD) | RF (Framingham) | EV | 257314 | 17497 (6.80) | 0.855 | 0.852 | 0.858 |  |  |
| Li 2022 (CPRD) | RF (ASSIGN) | IV | 838961 | 57049 (6.80) | 0.859 | 0.856 | 0.862 |  |  |
| Li 2022 (CPRD) | RF (ASSIGN) | EV | 257314 | 17497 (6.80) | 0.853 | 0.850 | 0.856 |  |  |
| Vu 2024 (Suita) | LR | D | 7389 | 438 (5.93) | 0.68 | 0.56 | 0.74 |  |  |
| Vu 2024 (Suita) | SVM | D | 7389 | 438 (5.93) | 0.73 | 0.67 | 0.79 |  |  |
| Vu 2024 (Suita) | RF | D | 7389 | 438 (5.93) | 0.71 | 0.65 | 0.77 |  |  |
| Vu 2024 (Suita) | XGBoost | D | 7389 | 438 (5.93) | 0.71 | 0.65 | 0.77 |  |  |
| Vu 2024 (Suita) | LightGBM | D | 7389 | 438 (5.93) | 0.7 | 0.64 | 0.76 |  |  |

## Supplementary Table S3. Prediction models and outcome definitions

| **Model (author)** | **Outcome definition** |
| --- | --- |
| Suita | Any stroke |
| PROCAM-stroke | Ischaemic only |
| Borglykke | Any stroke |
| Framingham | Any stroke |
| Chambless | Ischaemic only |
| Clinical (Chien) | Any stroke |
| Biochemical (Chien) | Any stroke |
| Clinial co-efficient based (Chien) | Any stroke |
| Biochemical co-efficient based (Chien) | Any stroke |
| R-FSRS | Any stroke |
| R-FSRS recalibrated (Chun) | Any stroke |
| R-FSRS refitted (Chun) | Any stroke |
| R-FSRS recalibrated by geographical area (Chun) | Any stroke |
| Reference model (Di Castelnuovo) | Any stroke |
| Basic IS (Ferket) | Ischaemic only |
| Basic - any stroke (Ferket) | Any stroke |
| PCE | Any stroke |
| CVD score | Any stroke |
| Harada - no AF | Any stroke |
| Harada - AF | Any stroke |
| Basic ACVD model | Any stroke |
| SRSRF (Howard) | Any stroke |
| DNN (Hung 2019) | Ischaemic only |
| MGL (Hung 2018) | Ischaemic only |
| SDA (Hung 2018) | Ischaemic only |
| ADA-L (Hung 2018) | Ischaemic only |
| ADA-M (Hung 2018) | Ischaemic only |
| ADA-S (Hung 2018) | Ischaemic only |
| Model all ages (Hunter) | Ischaemic only |
| Model <50 (Hunter) | Ischaemic only |
| Model- 50-59 (Hunter) | Ischaemic only |
| Model-60-69 (Hunter) | Ischaemic only |
| Model-70+ (Hunter) | Ischaemic only |
| TRS Model 1 (Jung 2018) | Any stroke |
| 5-year model (Lee S) | Any stroke |
| XGBoost (Li X 2022) | Any stroke |
| LR (Li X 2022) | Ischaemic only |
| SVM (Li X 2022) | Ischaemic only |
| RF (Li X 2022) | Ischaemic only |
| BEHRT (Li Y 2022) | Any stroke |
| Hi-BEHRT (Li Y 2022) | Any stroke |
| LR (Lolak) | Any stroke |
| BN (Lolak) | Any stroke |
| TAN (Lolak) | Any stroke |
| XGBoost (Lolak) | Any stroke |
| EBM (Lolak) | Any stroke |
| PRIME local (Majed) | Any stroke |
| Framingham calibrated (Majed) | Any stroke |
| Model A men (Marrugat) | Any stroke |
| Model A women (Marrugat) | Any stroke |
| Model B men (Marrugat) | Any stroke |
| Model B women (Marrugat) | Any stroke |
| Model 1 GRU (Teoh) | Any stroke |
| Model 2 CNN/RNN (Teoh) | Any stroke |
| Model 3 Dual (Teoh) | Any stroke |
| MetS 10 years (Wannamethee) | Any stroke |
| MetS 20 years (Wannamethee) | Any stroke |
| Final model (Yatsuya 2016) | Any stroke |
| Cox model (Yatsuya 2013) | Any stroke |
| Point-based model (Yatsuya 2013) | Any stroke |
| IS (Zhang X 2005) | Ischaemic only |
| Recalibrated R-FSRS (Zhang Y 2020) | Any stroke |
| Adjusted R-FSRS (Zhang Y 2020) | Any stroke |
| DNN (Hung 2017) | Ischaemic only |
| GBDT (Hung 2017) | Ischaemic only |
| Conventional risk factors men (Sun 2021) | Any stroke |
| Conventional risk factors women (Sun 2021) | Any stroke |
| Conventional risk factors + CRP men (Sun 2021) | Any stroke |
| Conventional risk factors + CRP women (Sun 2021) | Any stroke |
| Own model men (d’Agostino) | Any stroke |
| Own model women (d’Agostino) | Any stroke |
| BEHRT (Li 2022) | Any stroke |
| RF QRISK (Li 2022) | Any stroke |
| RF Framingham (Li 2022) | Any stroke |
| RF ASSIGN (Li 2022) | Any stroke |
| LR (Vu 2024) | Any stroke |
| SVM (Vu 2024) | Any stroke |
| RF (Vu 2024) | Any stroke |
| XGBoost (Vu 2024) | Any stroke |
| LightGBM (Vu 2024) | Any stroke |

## Supplementary Table S4. Prediction models by type

| **Regression models** | |
| --- | --- |
| **Model (author)** | **Regression type** |
| Suita | Cox regression |
| PROCAM-stroke | Cox regression |
| Borglykke | Cox regression |
| Framingham | Cox regression |
| Chambless | Cox regression |
| Clinical (Chien) | Cox regression |
| Biochemical (Chien) | Cox regression |
| Clinial co-efficient based (Chien) | Cox regression |
| Biochemical co-efficient based (Chien) | Cox regression |
| R-FSRS | Cox regression |
| R-FSRS recalibrated (Chun) | Cox regression |
| R-FSRS refitted (Chun) | Cox regression |
| R-FSRS recalibrated by geographical area (Chun) | Cox regression |
| Reference model (Di Castelnuovo) | Cox regression |
| Basic IS (Ferket) | Cox regression |
| Basic - any stroke (Ferket) | Cox regression |
| PCE | Cox regression |
| CVD score | Cox regression |
| Harada - no AF | Poisson regression |
| Harada - AF | Poisson regression |
| Basic ACVD model (Hilvo) | Cox regression |
| SRSRF (Howard) | Cox regression |
| Model all ages (Hunter) | Logistic regression |
| Model <50 (Hunter) | Logistic regression |
| Model- 50-59 (Hunter) | Logistic regression |
| Model-60-69 (Hunter) | Logistic regression |
| Model-70+ (Hunter) | Logistic regression |
| TRS Model 1 (Jung 2018) | Cox regression |
| 5-year model (Lee S) | Cox regression |
| PRIME local (Majed) | Cox regression |
| Framingham calibrated (Majed) | Cox regression |
| Model A men (Marrugat) | Cox regression |
| Model A women (Marrugat) | Cox regression |
| Model B men (Marrugat) | Cox regression |
| Model B women (Marrugat) | Cox regression |
| LR (Li X 2022) | Logistic regression |
| LR (Lolak) | Logistic regression |
| MetS 10 years (Wannamethee) | Cox regression |
| MetS 20 years (Wannamethee) | Cox regression |
| Final model (Yatsuya 2016) | Logistic regression |
| Cox model (Yatsuya 2013) | Cox regression |
| Point-based model (Yatsuya 2013) | Cox regression |
| IS (Zhang X 2005) | Cox regression |
| Recalibrated R-FSRS (Zhang Y 2020) | Cox regression |
| Adjusted R-FSRS (Zhang Y 2020) | Cox regression |
| Conventional risk factors men (Sun 2021) | Cox regression |
| Conventional risk factors women (Sun 2021) | Cox regression |
| Conventional risk factors + CRP men (Sun 2021) | Cox regression |
| Conventional risk factors + CRP women (Sun 2021) | Cox regression |
| Own model men (d’Agostino) | Cox regression |
| Own model women (d’Agostino) | Cox regression |
| **Machine learning models** | |
| **Model (author)** | **Machine learning type** |
| DNN (Hung 2019) | Neural network |
| MGL (Hung 2018) | Graph based model |
| SDA (Hung 2018) | Sparse autoencoder |
| ADA-L (Hung 2018) | Ensemble |
| ADA-M (Hung 2018) | Ensemble |
| ADA-S (Hung 2018) | Ensemble |
| XGBoost (Li X 2022) | Boosting |
| SVM (Li X 2022) | Support Vector Machine |
| RF (Li X 2022) | Random Forest |
| BEHRT (Li Y 2022) | Transformer-based |
| Hi-BEHRT (Li Y 2022) | Transformer-based |
| BN (Lolak) | Bayesian network |
| TAN (Lolak) | Tree-augmented Naïve Bayes |
| XGBoost (Lolak) | Boosting |
| EBM (Lolak) | Explainable boosting machine |
| Model 1 GRU (Teoh) | Recurrent neural network |
| Model 2 CNN/RNN (Teoh) | Hybrid (CNN/RNN) |
| Model 3 Dual (Teoh) | Neural network |
| DNN (Hung 2017) | Neural network |
| GBDT (Hung 2017) | Boosting |
| BEHRT (Li 2022) | Transformer-based |
| RF QRISK (Li 2022) | Random Forest |
| RF Framingham (Li 2022) | Random Forest |
| RF ASSIGN (Li 2022) | Random Forest |
| LR (Vu 2024) | Linear regression |
| SVM (Vu 2024) | Support vector machine |
| RF (Vu 2024) | Random Forest |
| XGBoost (Vu 2024) | Boosting |
| LightGBM (Vu 2024) | Light Gradient-Boosting Machine |

## Supplementary Table S5: Baseline variables used in regression models

| **Model / Study** | **Predictors** | | | | |
| --- | --- | --- | --- | --- | --- |
|  | **Patient characteristics** | **Medical history** | **Physical measurements** | **Investigations** | **Other** |
| Suita | Age, smoking | DM, CKD, AF | SBP | Blood glucose |  |
| PROCAM-stroke | Age, sex, smoking | DM | SBP |  |  |
| Borlgykke *et al* 2010 | Sex, smoking | Diabetes, HTN | BMI, SBP, DBP | Cholesterol - total, HDL |  |
| Framingham | Smoking | HTN, DM | SBP, BMI | Total cholesterol, HDL | Medications: antihypertensive |
| Chambless *et al* 2004 | Smoking | DM, HTN, coronary disease | SBP |  |  |
| Chien *et al* 2010 | Age, sex | AF, DM | SBP, DBP |  | Family history of stroke |
| R-FSRS | Age, smoking | CHD, DM, HTN | SBP |  |  |
| Di Castelnuovo *et al* 2019 - Reference model | Age, sex, smoking | DM, HTN, MI | BMI, SBP, DBP | Total cholesterol, HDL |  |
| Ferket *et al* 2014 | Age, sex, ethnicity, smoking | DM, HTN, coronary disease | SBP |  |  |
| PCE | Age, sex, race, smoking | HTN, DM | SBP | Total cholesterol, HDL |  |
| Foraker *et al* 2016 – CVD score | Age, sex, race, smoking | HTN, DM | SBP | Cholesterol | Cholesterol treatment |
| Harada *et al* 2018 - no AF | Age, sex, smoking | DM | SBP | HDL |  |
| Harada et al 2018 - AF | Age, sex, smoking | AF, DM | SBP | HDL |  |
| Hilvo *et al* 2022 - basic ACVD | Age, sex, smoking |  | SBP | Total cholesterol |  |
| Hunter *et al* 2022 | Sex, smoking | AF, DM, HTN | BMI, SBP, DBP | Total cholesterol |  |
| Jung *et al* 2018 - TRS model | Age, sex, smoking | HTN, DM, dyslipidaemia |  |  |  |
| Majed *et al* 2013 – PRIME | Age, sex, smoking | DM | BP | Total cholesterol, HDL |  |
| Framingham | Age, sex, smoking | DM | BP | Total cholesterol, HDL |  |
| Marrugat *et al* 2014 - model A | Age, smoking |  | BMI |  |  |
| Marrugat et al 2014 - model B | Age, smoking | DM, HTN | BMI, SBP | Total cholesterol, HDL | Medications: antihypertensive |
| Wannamethee *et al* 2005 - MetS |  | HTN | BP, BMI | Fasting glucose, triglycerides, HDL | Medications: antihypertensive |
| Yatsuya *et al* 2016 | Age, sex, smoking | DM, HTN | SBP | HDL | Medications: antihypertensive |
| Yatsuya *et al* 2013 | Age, sex, smoking, alcohol | DM, HTN | BMI, BP |  | Medication: antihypertensive |
| Zhang X *et al* 2005 - IS | Age, smoking |  | BP | Total cholesterol |  |
| Sun *et al* 2021 – conventional | Age, sex, smoking | DM | SBP | Total cholesterol, HDL |  |
| Sun *et al* 2021 – conventional + CRP | Age, sex, smoking | DM | SBP | Total cholesterol, HDL, CRP |  |
| Framingham Wilson | Age, smoking | DM | Untreated SBP, treated SBP | Total cholesterol, HDL |  |
| D’Agostino *et al 2008* | Age, smoking | DM, HTN | SBP | Total cholesterol, HDL | Medication: antihypertensive |

Abbreviations: AF, atrial fibrillation; BMI, body mass index; BP, blood pressure; CHD coronary heart disease; CKD, chronic kidney disease; CRP, C-reactive protein; DBP, diastolic blood pressure; DM, diabetes mellitus; HDL, high density lipoprotein; HTN, hypertension; SBP, systolic blood pressure

## Supplementary Table S6: Baseline variables used in machine learning models

|  | **Variable category** | | | | | | | |
| --- | --- | --- | --- | --- | --- | --- | --- | --- |
| **Study (models)** | **Diagnoses** | **Diagnostics** | **Medications** | **Hospitalisation** | **Clinical features** | **Demographics** | **Vital signs** | **Treatments/**  **procedures** |
| Hung *et al* 2019 (DNN) | X |  | X |  |  | X |  |  |
| Hung *et al* 2018 (MGL, SDA, ADA-L, ADA-M, ADA-S)* |  |  |  |  |  |  |  |  |
| Li X *et al* 2022 (XGBoost, LR, SVM, RF) |  | X |  |  |  | X |  |  |
| Li Y *et al* 2022 (BEHRT, Hi-BEHRT) | X | X | X |  |  | X | X |  |
| Lolak *et al* (BN, TAN, XGBoost, EBM) | X | X | X |  | X | X | X |  |
| Teoh *et al* (GRU, CNN/RNN, Dual) | X |  | X |  |  |  | X |  |
| Hung *et al* 2017 (DNN, GBDT) | X |  | X |  |  | X |  |  |
| Li *et al* 2022 (BEHRT) | X | X |  |  |  | X | X |  |
| Vu *et al* 2024 | X | X | X |  |  | X | X |  |

*Variables not reported

## Supplementary Table S7: Risk of bias and applicability assessment for each PROBAST domain

| **Study** | **ROB Participants** | **ROB Predictors** | **ROB Outcomes** | **ROB Analysis** | **Applicability Participants** | **Applicability Predictors** | **Applicability Outcomes** | **ROB Overall** | **Applicability Overall** |
| --- | --- | --- | --- | --- | --- | --- | --- | --- | --- |
| Arafa et al 2021 |  |  |  |  |  |  |  |  |  |
| Assman et al 2007 |  |  |  |  |  |  |  |  |  |
| Borglykke et al 2010 |  |  |  |  |  |  |  |  |  |
| Camen et al 2020 |  |  |  |  |  |  |  |  |  |
| Chambless et al 2004 |  |  |  |  |  |  |  |  |  |
| Chien et al 2010 |  |  |  |  |  |  |  |  |  |
| Chun et al 2022 |  |  |  |  |  |  |  |  |  |
| Di Castelnuovo et al 2019 |  |  |  |  |  |  |  |  |  |
| Ferket et al 2014 |  |  |  |  |  |  |  |  |  |
| Flueckiger et al 2018 |  |  |  |  |  |  |  |  |  |
| Foraker et al 2016 |  |  |  |  |  |  |  |  |  |
| Harada et al 2018 |  |  |  |  |  |  |  |  |  |
| Hilvo et al 2022 |  |  |  |  |  |  |  |  |  |
| Hong et al 2023 |  |  |  |  |  |  |  |  |  |
| Howard et al 2017 |  |  |  |  |  |  |  |  |  |
| Hung et al 2019 |  |  |  |  |  |  |  |  |  |
| Hung et al 2018 |  |  |  |  |  |  |  |  |  |
| Hunter et al 2022 |  |  |  |  |  |  |  |  |  |
| Jung et al 2018 |  |  |  |  |  |  |  |  |  |
| Lee et al 2020 |  |  |  |  |  |  |  |  |  |
| Li X et al 2022 |  |  |  |  |  |  |  |  |  |
| Li Y et al 2022 (1) |  |  |  |  |  |  |  |  |  |
| Lolak et al 2023 |  |  |  |  |  |  |  |  |  |
| Majed et al 2013 |  |  |  |  |  |  |  |  |  |
| Marrugat et al 2014 |  |  |  |  |  |  |  |  |  |
| Teoh et al 2018 |  |  |  |  |  |  |  |  |  |
| Wannamethee et al 2005 |  |  |  |  |  |  |  |  |  |
| Wu et al 2020 |  |  |  |  |  |  |  |  |  |
| Yang et al 2023 |  |  |  |  |  |  |  |  |  |
| Yatsuya et al 2016 |  |  |  |  |  |  |  |  |  |
| Yatsuya et al 2013 |  |  |  |  |  |  |  |  |  |
| Zhang X et al 2005 |  |  |  |  |  |  |  |  |  |
| Zhang Y et al 2020 |  |  |  |  |  |  |  |  |  |
| Dufouil et al 2017 |  |  |  |  |  |  |  |  |  |
| Hung et al 2017 |  |  |  |  |  |  |  |  |  |
| Sun et al 2021 |  |  |  |  |  |  |  |  |  |
| Xing et al 2019 |  |  |  |  |  |  |  |  |  |
| Bos et al 2017 |  |  |  |  |  |  |  |  |  |
| D’Agostino et al 2008 |  |  |  |  |  |  |  |  |  |
| Li Y et al 2022 (2) |  |  |  |  |  |  |  |  |  |
| Vu et al 2024 |  |  |  |  |  |  |  |  |  |

## Supplementary Figure S1: Sensitivity analysis for prediction models after excluding studies with high risk of bias and excluding derivation cohorts


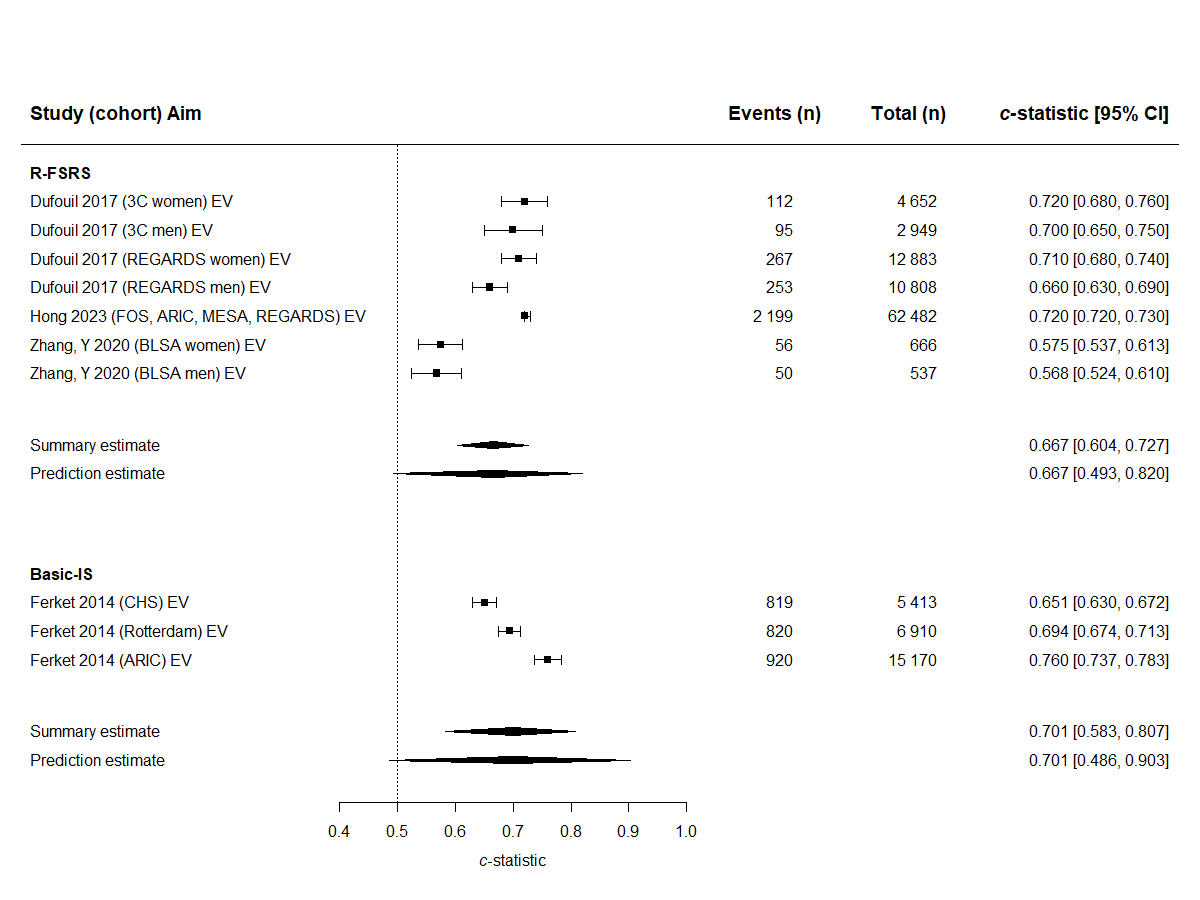


## Supplementary Figure S2: Analysis of R-FSRS according to sex


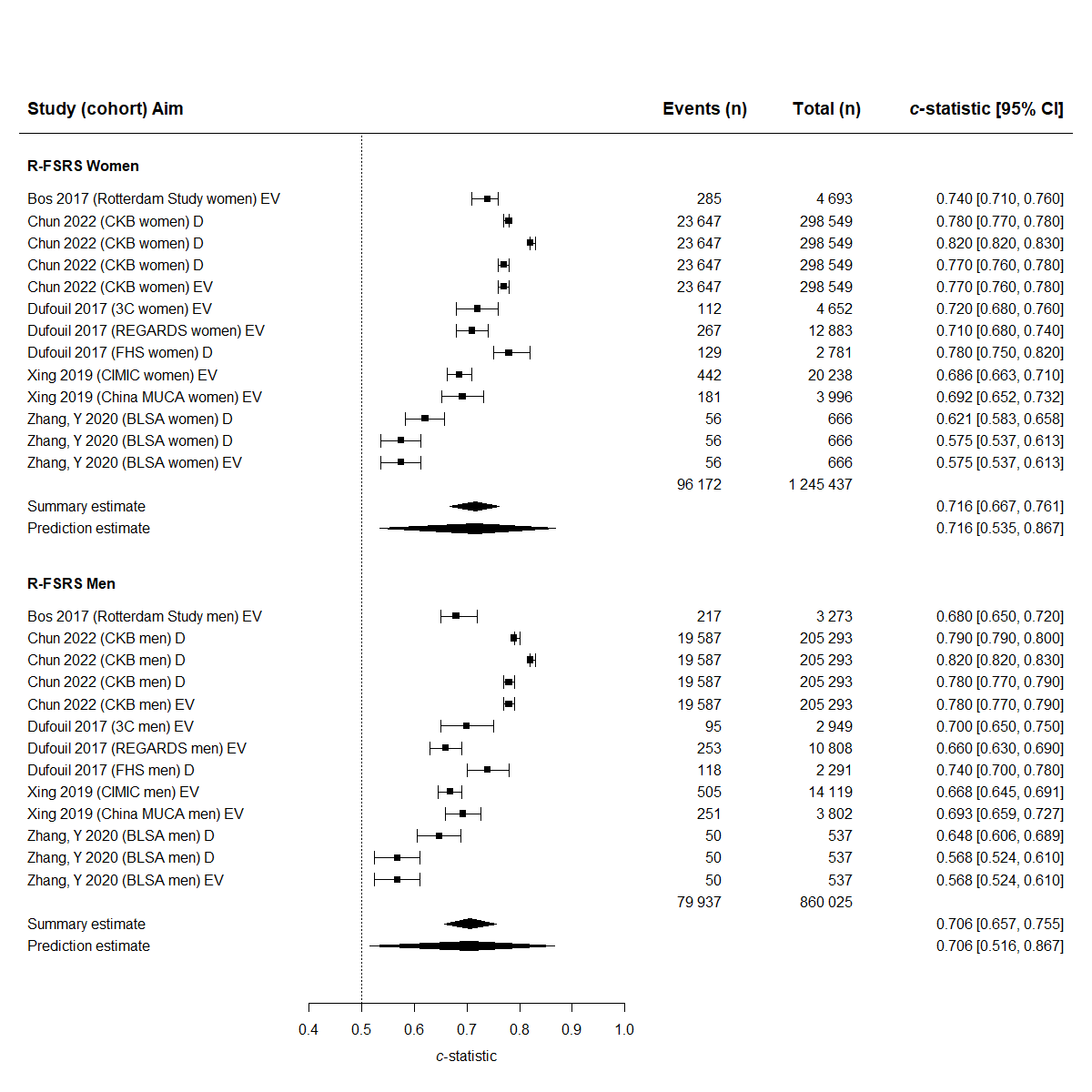


## Supplementary Figure S3: Forest plot of regression and machine learning models


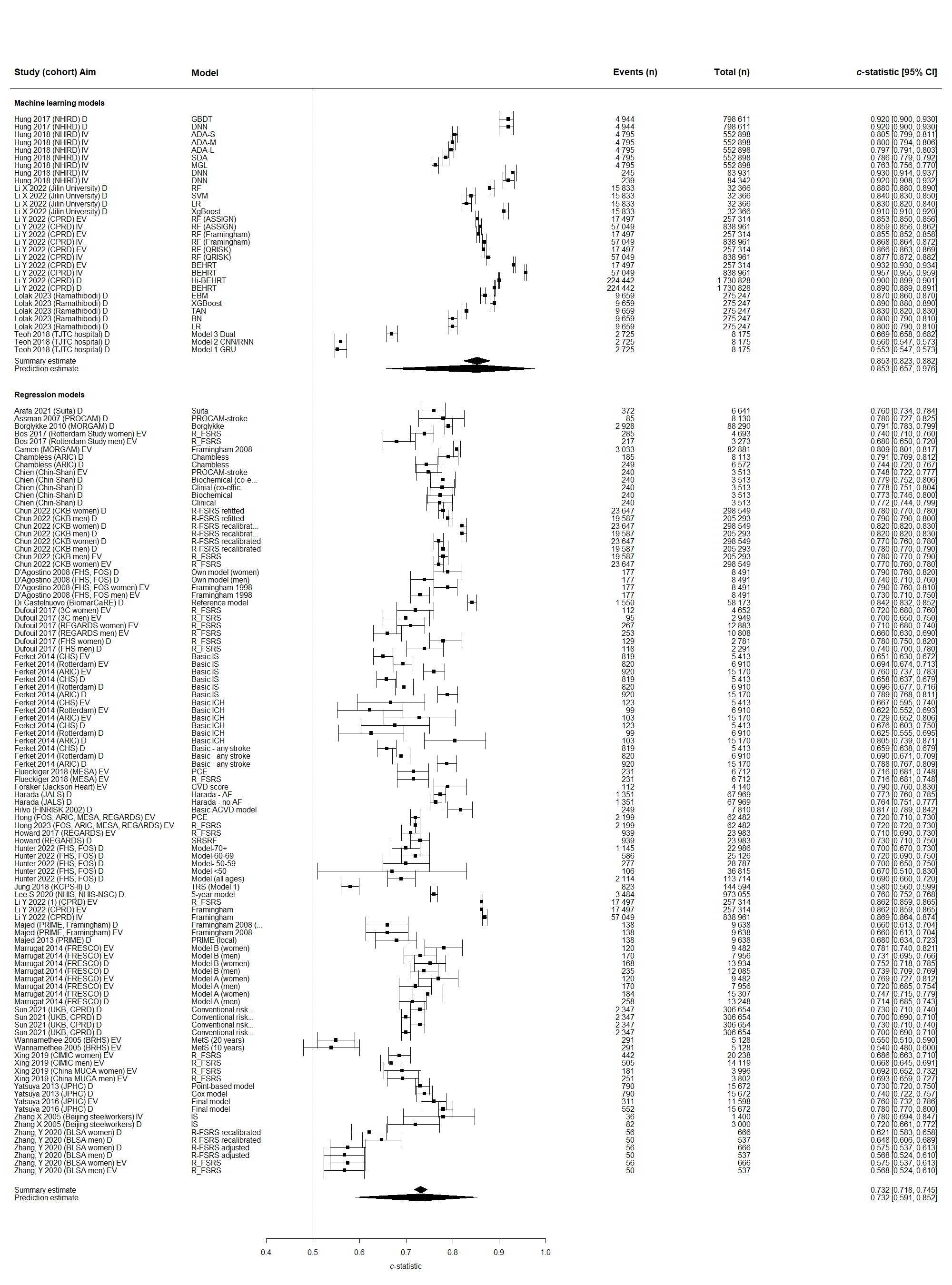


## Supplementary Figure S4: Funnel plot


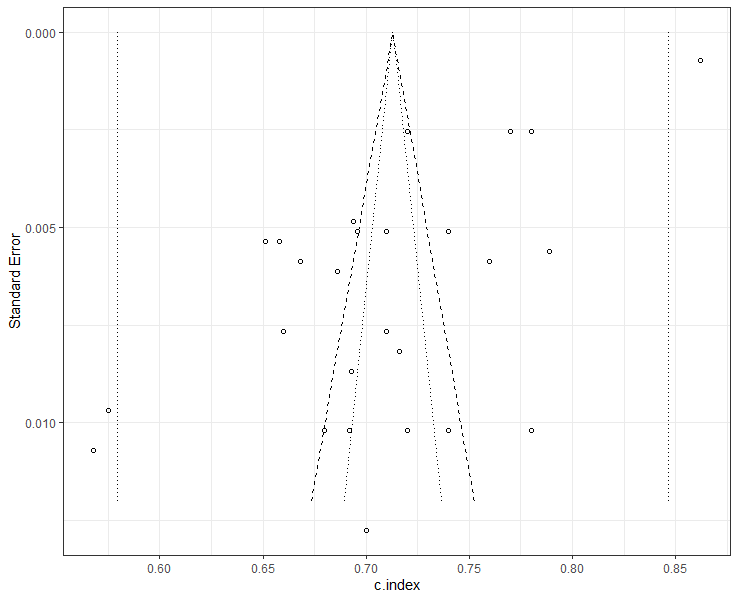


# PRISMA CHECKLIST

| **Section and Topic** | **Item #** | **Checklist item** | **Location where item is reported** |
| --- | --- | --- | --- |
| **TITLE** | | |  |
| Title | 1 | Identify the report as a systematic review. | 1 |
| **ABSTRACT** | | |  |
| Abstract | 2 | See the PRISMA 2020 for Abstracts checklist. | Abstract |
| **INTRODUCTION** | | |  |
| Rationale | 3 | Describe the rationale for the review in the context of existing knowledge. | 5 |
| Objectives | 4 | Provide an explicit statement of the objective(s) or question(s) the review addresses. | 5 |
| **METHODS** | | |  |
| Eligibility criteria | 5 | Specify the inclusion and exclusion criteria for the review and how studies were grouped for the syntheses. | 6 |
| Information sources | 6 | Specify all databases, registers, websites, organisations, reference lists and other sources searched or consulted to identify studies. Specify the date when each source was last searched or consulted. | 6 |
| Search strategy | 7 | Present the full search strategies for all databases, registers and websites, including any filters and limits used. | Supplementary material |
| Selection process | 8 | Specify the methods used to decide whether a study met the inclusion criteria of the review, including how many reviewers screened each record and each report retrieved, whether they worked independently, and if applicable, details of automation tools used in the process. | 6 |
| Data collection process | 9 | Specify the methods used to collect data from reports, including how many reviewers collected data from each report, whether they worked independently, any processes for obtaining or confirming data from study investigators, and if applicable, details of automation tools used in the process. | 6-7 |
| Data items | 10a | List and define all outcomes for which data were sought. Specify whether all results that were compatible with each outcome domain in each study were sought (e.g. for all measures, time points, analyses), and if not, the methods used to decide which results to collect. | 7-8 |
|  | 10b | List and define all other variables for which data were sought (e.g. participant and intervention characteristics, funding sources). Describe any assumptions made about any missing or unclear information. | 7-8 |
| Study risk of bias assessment | 11 | Specify the methods used to assess risk of bias in the included studies, including details of the tool(s) used, how many reviewers assessed each study and whether they worked independently, and if applicable, details of automation tools used in the process. | 7 |
| Effect measures | 12 | Specify for each outcome the effect measure(s) (e.g. risk ratio, mean difference) used in the synthesis or presentation of results. | 7-8 |
| Synthesis methods | 13a | Describe the processes used to decide which studies were eligible for each synthesis (e.g. tabulating the study intervention characteristics and comparing against the planned groups for each synthesis (item #5)). | 7-8 |
|  | 13b | Describe any methods required to prepare the data for presentation or synthesis, such as handling of missing summary statistics, or data conversions. | 8 |
|  | 13c | Describe any methods used to tabulate or visually display results of individual studies and syntheses. | 8 |
|  | 13d | Describe any methods used to synthesize results and provide a rationale for the choice(s). If meta-analysis was performed, describe the model(s), method(s) to identify the presence and extent of statistical heterogeneity, and software package(s) used. | 8 |
|  | 13e | Describe any methods used to explore possible causes of heterogeneity among study results (e.g. subgroup analysis, meta-regression). | 8 |
|  | 13f | Describe any sensitivity analyses conducted to assess robustness of the synthesized results. | 8 |
| Reporting bias assessment | 14 | Describe any methods used to assess risk of bias due to missing results in a synthesis (arising from reporting biases). | 8 |
| Certainty assessment | 15 | Describe any methods used to assess certainty (or confidence) in the body of evidence for an outcome. | 8-9 |
| **RESULTS** | | |  |
| Study selection | 16a | Describe the results of the search and selection process, from the number of records identified in the search to the number of studies included in the review, ideally using a flow diagram. | 10, Figure 1 |
|  | 16b | Cite studies that might appear to meet the inclusion criteria, but which were excluded, and explain why they were excluded. | Supplementary material |
| Study characteristics | 17 | Cite each included study and present its characteristics. | Table 1, Table S1, supplementary material |
| Risk of bias in studies | 18 | Present assessments of risk of bias for each included study. | Figure 3, Table S6 |
| Results of individual studies | 19 | For all outcomes, present, for each study: (a) summary statistics for each group (where appropriate) and (b) an effect estimate and its precision (e.g. confidence/credible interval), ideally using structured tables or plots. | 11-12, Table S1 |
| Results of syntheses | 20a | For each synthesis, briefly summarise the characteristics and risk of bias among contributing studies. | 11-12 |
|  | 20b | Present results of all statistical syntheses conducted. If meta-analysis was done, present for each the summary estimate and its precision (e.g. confidence/credible interval) and measures of statistical heterogeneity. If comparing groups, describe the direction of the effect. | 11-12, Figure 4, S3 |
|  | 20c | Present results of all investigations of possible causes of heterogeneity among study results. | N/A |
|  | 20d | Present results of all sensitivity analyses conducted to assess the robustness of the synthesized results. | Figure S1, S2 |
| Reporting biases | 21 | Present assessments of risk of bias due to missing results (arising from reporting biases) for each synthesis assessed. | N/A |
| Certainty of evidence | 22 | Present assessments of certainty (or confidence) in the body of evidence for each outcome assessed. | 12 |
| **DISCUSSION** | | |  |
| Discussion | 23a | Provide a general interpretation of the results in the context of other evidence. | 13-15 |
|  | 23b | Discuss any limitations of the evidence included in the review. | 14-15 |
|  | 23c | Discuss any limitations of the review processes used. | 14-15 |
|  | 23d | Discuss implications of the results for practice, policy, and future research. | 13-15 |
| **OTHER INFORMATION** | | |  |
| Registration and protocol | 24a | Provide registration information for the review, including register name and registration number, or state that the review was not registered. | 6 |
|  | 24b | Indicate where the review protocol can be accessed, or state that a protocol was not prepared. | 6 |
|  | 24c | Describe and explain any amendments to information provided at registration or in the protocol. | 6 |
| Support | 25 | Describe sources of financial or non-financial support for the review, and the role of the funders or sponsors in the review. | 17 |
| Competing interests | 26 | Declare any competing interests of review authors. | 17 |
| Availability of data, code and other materials | 27 | Report which of the following are publicly available and where they can be found: template data collection forms; data extracted from included studies; data used for all analyses; analytic code; any other materials used in the review. | 17 |

# PRISMA ABSTRACT CHECKLIST

| **Section and Topic** | **Item #** | **Checklist item** | **Reported (Yes/No)** |
| --- | --- | --- | --- |
| **TITLE** | | |  |
| Title | 1 | Identify the report as a systematic review. | Yes |
| **BACKGROUND** | | |  |
| Objectives | 2 | Provide an explicit statement of the main objective(s) or question(s) the review addresses. | Yes |
| **METHODS** | | |  |
| Eligibility criteria | 3 | Specify the inclusion and exclusion criteria for the review. | Yes |
| Information sources | 4 | Specify the information sources (e.g. databases, registers) used to identify studies and the date when each was last searched. | Yes |
| Risk of bias | 5 | Specify the methods used to assess risk of bias in the included studies. | Yes |
| Synthesis of results | 6 | Specify the methods used to present and synthesise results. | Yes |
| **RESULTS** | | |  |
| Included studies | 7 | Give the total number of included studies and participants and summarise relevant characteristics of studies. | Yes |
| Synthesis of results | 8 | Present results for main outcomes, preferably indicating the number of included studies and participants for each. If meta-analysis was done, report the summary estimate and confidence/credible interval. If comparing groups, indicate the direction of the effect (i.e. which group is favoured). | Yes |
| **DISCUSSION** | | |  |
| Limitations of evidence | 9 | Provide a brief summary of the limitations of the evidence included in the review (e.g. study risk of bias, inconsistency and imprecision). | Yes |
| Interpretation | 10 | Provide a general interpretation of the results and important implications. | Yes |
| **OTHER** | | |  |
| Funding | 11 | Specify the primary source of funding for the review. | Yes |
| Registration | 12 | Provide the register name and registration number. | Yes |

# REFERENCES

1. Ammenwerth E, Neyer S, Hörbst A, Mueller G, Siebert U, Schnell-Inderst P. Adult patient access to electronic health records. Cochrane Database Syst Rev. 2021 Feb 26;2(2):CD012707.

2. Poorthuis MHF, Jones NR, Sherliker P, Clack R, de Borst GJ, Clarke R, et al. Utility of risk prediction models to detect atrial fibrillation in screened participants. Eur J Prev Cardiol. 2021 May 22;28(6):586–95.

3. Nadarajah R, Alsaeed E, Hurdus B, Aktaa S, Hogg D, Bates MGD, et al. Prediction of incident atrial fibrillation in community-based electronic health records: a systematic review with meta-analysis. Heart. 2022 Jun 10;108(13):1020–9.

4. Moons KGM, Wolff RF, Riley RD, Whiting PF, Westwood M, Collins GS, et al. PROBAST: A Tool to Assess Risk of Bias and Applicability of Prediction Model Studies: Explanation and Elaboration. Ann Intern Med. 2019 Jan 1;170(1):W1–33.

5. Snell KI, Ensor J, Debray TP, Moons KG, Riley RD. Meta-analysis of prediction model performance across multiple studies: Which scale helps ensure between-study normality for the C-statistic and calibration measures? Stat Methods Med Res. 2018 Nov;27(11):3505–22.

6. Debray T, De Jong V. metamisc: Meta-Analysis of Diagnosis and Prognosis Research Studies [Internet]. 2012 [cited 2024 Jul 16]. p. 0.4.0. Available from: https://CRAN.R-project.org/package=metamisc

7. Debray TP, Damen JA, Riley RD, Snell K, Reitsma JB, Hooft L, et al. A framework for meta-analysis of prediction model studies with binary and time-to-event outcomes. Stat Methods Med Res. 2019 Sep;28(9):2768–86.

8. Remoortel HV, Scheers H, Buck ED, Haenen W, Vandekerckhove P. Prediction modelling studies for medical usage rates in mass gatherings: A systematic review. PLOS ONE. 2020 Jun 23;15(6):e0234977.

9. Peduzzi P, Concato J, Feinstein AR, Holford TR. Importance of events per independent variable in proportional hazards regression analysis. II. Accuracy and precision of regression estimates. J Clin Epidemiol. 1995 Dec;48(12):1503–10.

10. van der Ploeg T, Austin PC, Steyerberg EW. Modern modelling techniques are data hungry: a simulation study for predicting dichotomous endpoints. BMC Med Res Methodol. 2014 Dec 22;14:137.

11. Collins GS, Ogundimu EO, Altman DG. Sample size considerations for the external validation of a multivariable prognostic model: a resampling study. Stat Med. 2016 Jan 30;35(2):214–26.

12. Pencina MJ, D’Agostino RB. Evaluating Discrimination of Risk Prediction Models: The C Statistic. JAMA. 2015 Sep 8;314(10):1063–4.
